# Supplementary material for: Depletion-dependent activity-based protein profiling using SWATH/DIA-MS detects serine hydrolase lipid remodeling in lung adenocarcinoma progression
Source: Nat Commun. 2025 May 27;16:4889. doi: 10.1038/s41467-025-59564-x (PMC12117057; doi:10.1038/s41467-025-59564-x)
Supplement: Supplementary file 1 — Supplementary Information [file 41467_2025_59564_MOESM1_ESM.pdf]

Supplementary figures and legends

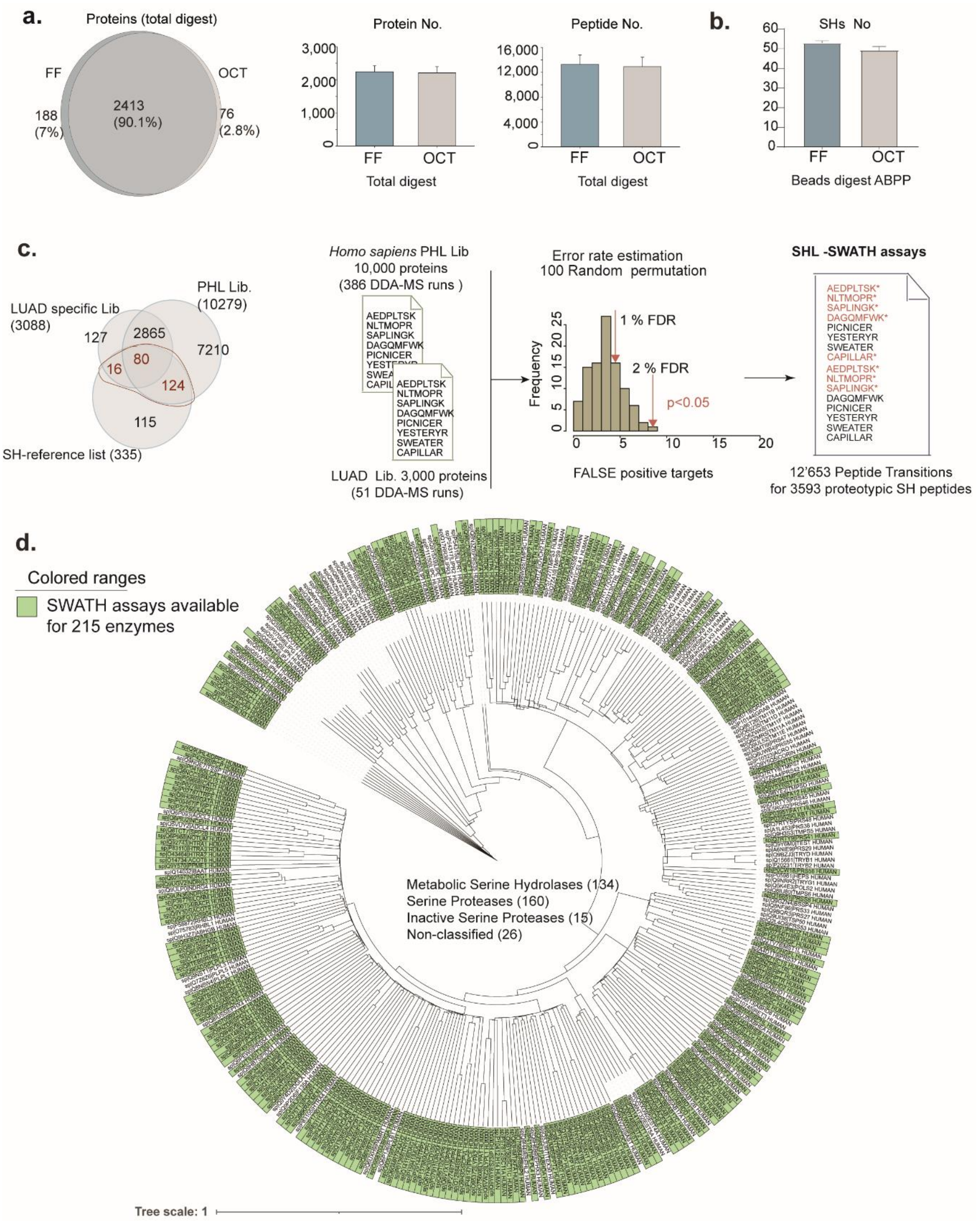

Supplementary Fig. 1. Comparison of dd-ABPP extraction protocol on frozen and OCT-embedded lung tissue and summary of SH-targeted DIA library (SHL) generation for human tissues.

a, Venn diagram shows overlap of protein identification between sample types, frozen (FF) and OCT-embedded lung tissue. Barplots with corresponding standard error (n=2-4) represent the number of proteins and peptides identified from FF and OCT-embedded tissue, respectively. **b**, Barplots with corresponding standard error (n=2-4) represent the number of captured enzymes in beads digest. **c**, Venn diagram shows overlap of SH identification (reference list of 335 SHs) between different human libraries. LC-MS peptide transition assays per SH protein were available from a deposited library of human tissue derived peptides (PHL or *Homo sapiens* library) or were generated from LUAD sample library (i.e., > 400 MS runs). The error rate distribution (i.e., number of FALSE positive (FP) targets) of new hybrid SHL was tested from 100 random combinations of two hypothetically created libraries of similar size to two combined libraries (adjusted to 1% FDR) and was estimated below 2% FDR. Final SHL library included 12653 peptide transition groups for 3593 proteotypic SH peptides (i.e. 215 enzymes, Supplementary Data 1). **d**, A phylogenetic tree of 327 out of 335 SH family members of which 294 (i.e. 160 serine proteases and 134 metabolic serine hydrolases) are annotated–, 15 human inactive–, and 26 non-annotated cases (non-classified cases, see Methods: phylogenetic tree). Light green boxes depict 64% (i.e., 215 proteins of which 191 annotated SH enzymes) of all cases for which LC-MS assays are available in SHL. Source data Figure 1.

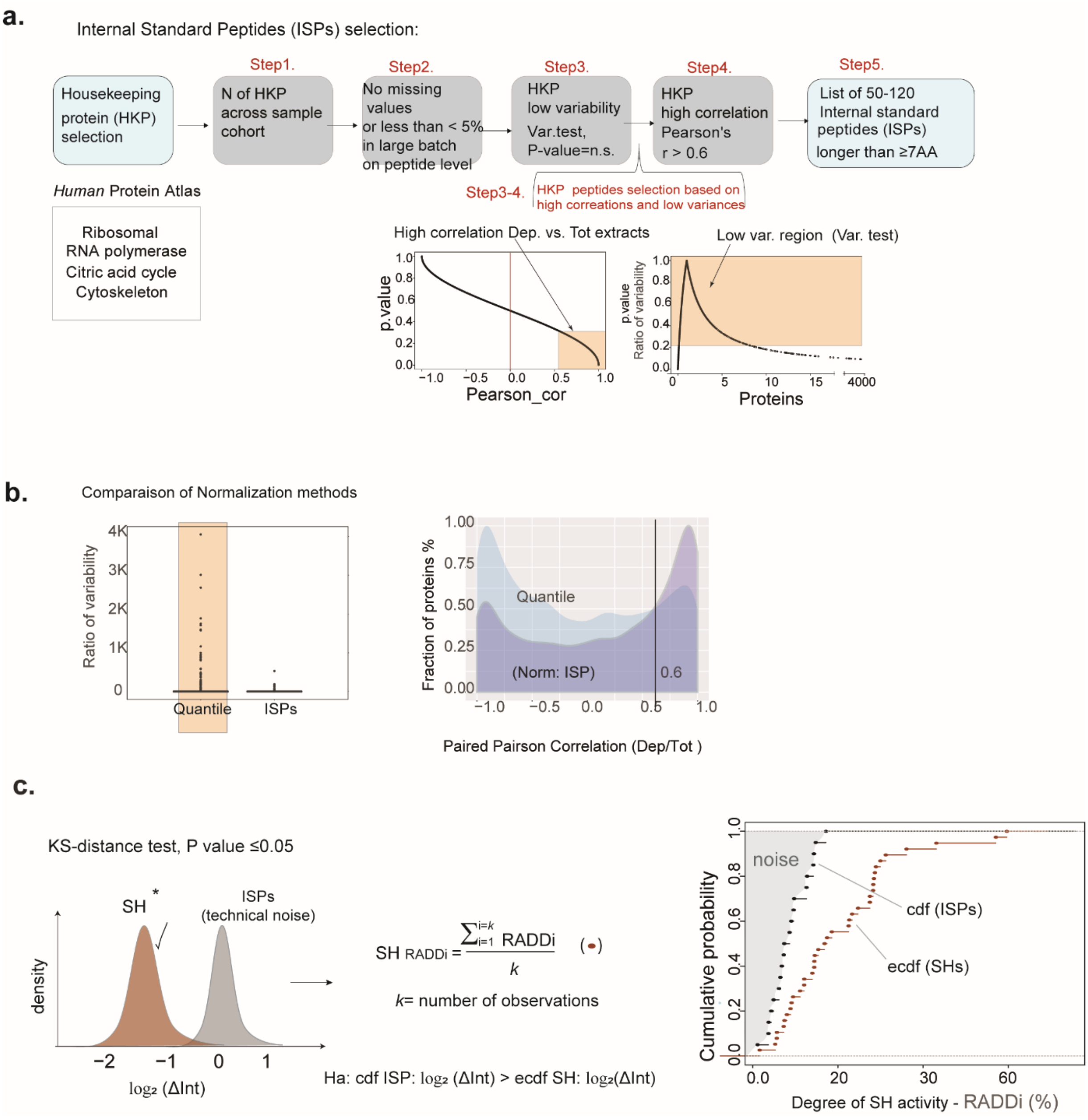

a.
Internal Standard Peptides (ISPs) selection:
Housekeeping protein (HKP) selection
Step1.
N of HKP across sample cohort
Step2.
No missing values or less than < 5% in large batch on peptide level
Step3.
HKP low variability Var.test, P-value=n.s.
Step4.
HKP high correlation Pearson's r > 0.6
Step5.
List of 50-120 Internal standard peptides (ISPs) longer than ≥7AA
Human Protein Atlas
Ribosomal
RNA polymerase
Citric acid cycle
Cytoskeleton
Step3-4.
HKP peptides selection based on high correations and low variances
High correlation Dep. vs. Tot extracts
p.value
Pearson_cor
Low var. region (Var. test)
p.value Ratio of variability
Proteins
b.
Comparaison of Normalization methods
Ratio of variability
Quantile
ISPs
Fraction of proteins %
Quantile
(Norm: ISP)
0.6
Paired Pairson Correlation (Dep/Tot)
c.
KS-distance test, P value ≤0.05
SH
ISPs (technical noise)
density
log2 (ΔInt)
SH RADDi = Σ i=1 to i=k RADDi / k
k= number of observations
Ha: cdf ISP: log2 (ΔInt) > ecdf SH: log2(ΔInt)
noise
cdf (ISPs)
ecdf (SHs)
Cumulative probability
Degree of SH activity - RADDi (%)

Supplementary Fig. 2. Procedure for normalization of measurement variability between total (Tot) and depleted (Dep) paired tissue extracts.

a, Five-fold selection criteria (step1-5) to obtain the list of stable endogenous ISPs (see Methods) of housekeeping (HK) and cytoskeletal (CS) proteins. In step 3-4, we aim to select the HK/CS proteins with low variability and high correlation between paired extracts. Density plot represents distribution of P-value (right panel) and Pearson correlation coefficient (Pearson cc test for paired samples, left panel) computed between paired extracts for each detected protein. P-value from F-test used to compare individual variances of proteins between Dep and Tot extracts (Ratio of variability, right panel). Orange box depicts area that corresponds to high correlations and low variances across paired extracts. **b**, ISPs reduce the technical noise variability between paired extracts of the same biological origin to optimize the detection of differences due to enzyme depletion. Comparison of ISP-normalization to Quantile. Left panel: Boxplot of variability ratio between Dep and Tot extract, after ISPs and Quantile normalization. Orange box depicts the higher protein variability (left) with Quantile normalization. Right panel: Density plots represent distribution of Pearson cc (paired) for each detected protein after ISPs (violet) and after Quantile (blue) normalization (right). **c**, The empirical depletion ratio of each detected SH enzyme was assessed from its SWATH/DIA-MS fragment ion intensity differences (i.e., log2ΔInt) of paired samples and was used to compute the enzyme RADDi. On the left: The Kolmogorov–Smirnov (KS) statistic with one-sided P-value ≤0.05 was used to select confident "active" SH enzymes. The distribution shift of empirical SH depletion ratio (log2ΔInt) was compared with ISPs reference distribution (technical noise) with an alternative hypothesis (Ha) that the cumulative distribution function (CDF) of ISP lies above that of empirical CDF of SH (Methods). On the right: The plot represents CDF of active enzyme fractions wherein each dot corresponds to respective SH average activity (i.e., RADDi) within the tested condition. The k corresponds to the number of observations (samples) per condition. Enzyme activity or RADDi value was set to 0 if its depletion value fell within the defined noise range (gray area).

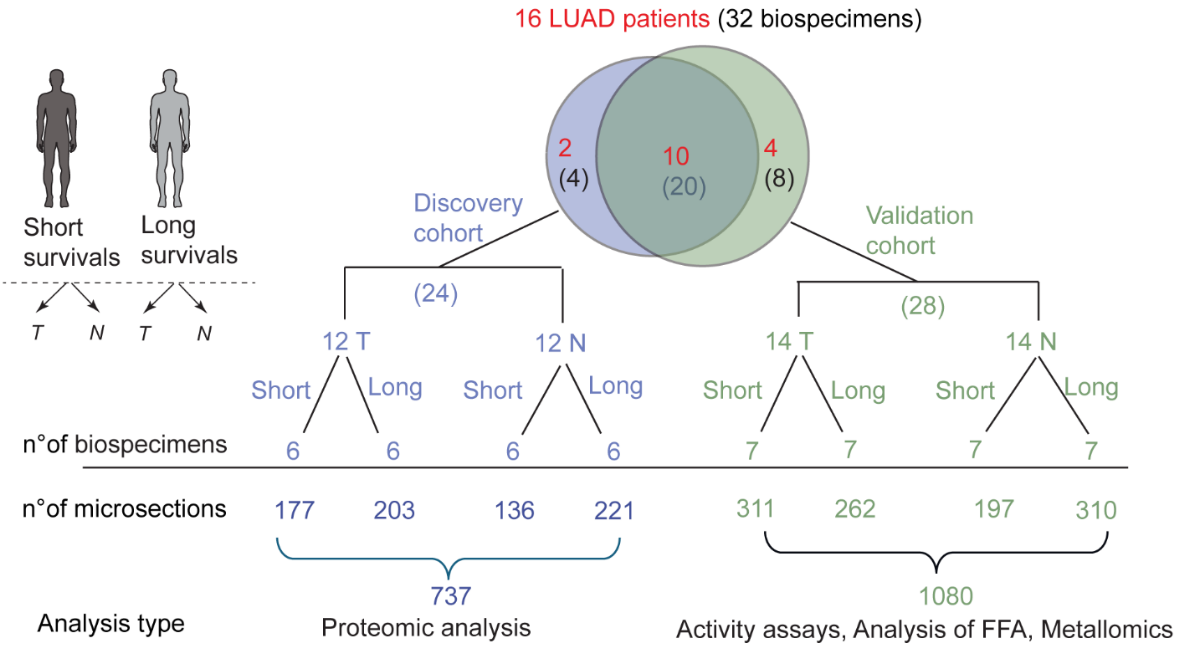

Supplementary Fig. 3. Number of patients, biospecimens, and microsections in each cohort, discovery cohort, and validation cohort.

Venn diagram depicting the number of patients, biospecimens, and microsections in the discovery cohort (left) and validation cohort (right). In total, we collected 32 tumors and adjacent tissues from 16 patients with LUAD type IIIA adenocarcinoma, 8 of which had only 1-year overall survival. For the initial proteomic analysis, we used 24 (out of 32) samples and processed 203 and 221 sections from 6 tumors and 6 non-tumors of long-term survival patients and 177 and 136 sections from 6 tumors and 6 non-tumors of short-term survival patients, respectively. For validation, we collected an additional set of 1080 cryostat sections covering 28 tumor and adjacent tissues from 14 LUAD patients, ten of which were also analyzed within the original discovery cohort and four were independent. The type of analysis performed on each sample set is reported.

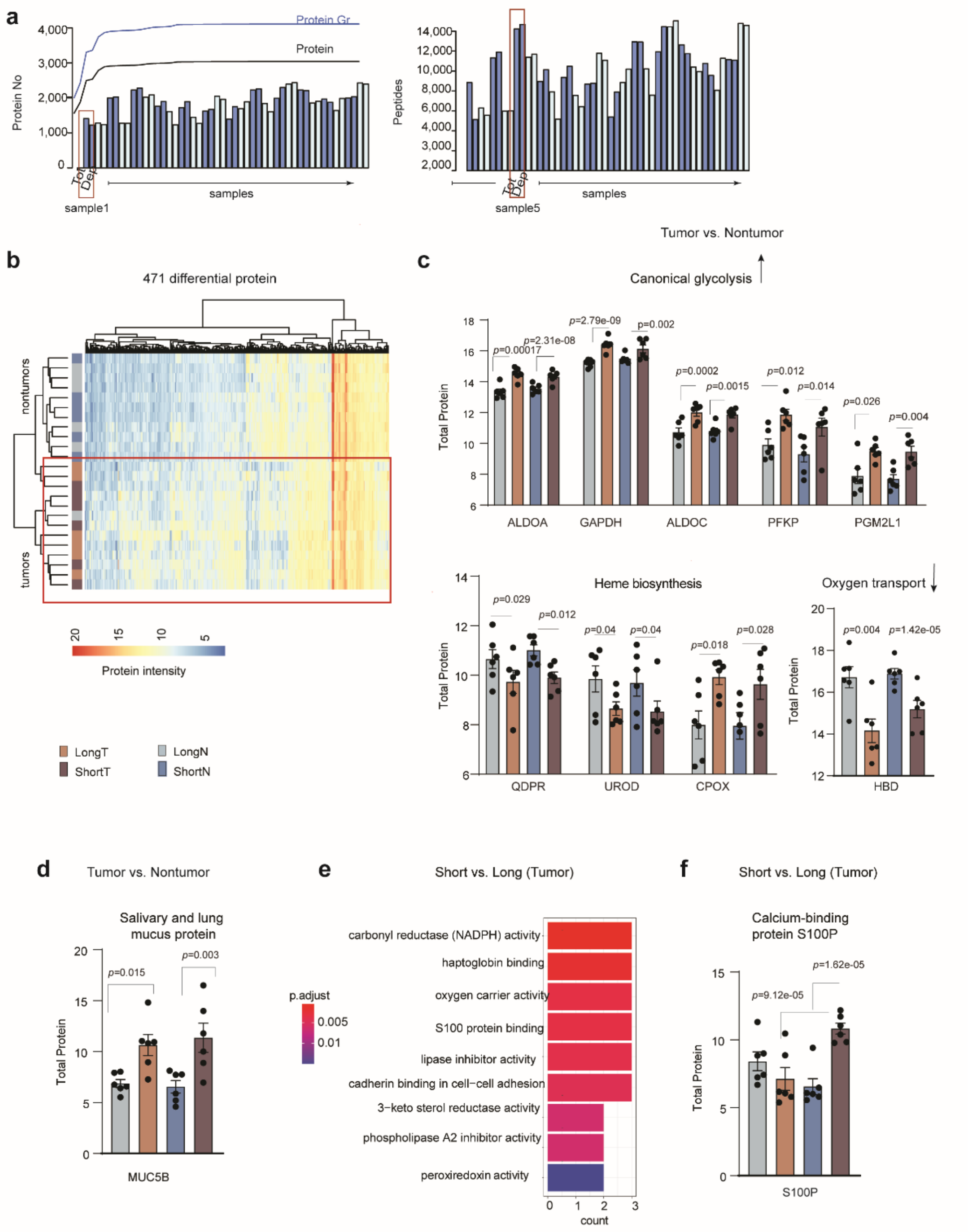
a
Protein No
Protein Gr
Protein
samples
sample1
Peptides
samples
sample5
b
471 differential protein
nontumors
tumors
Protein intensity
LongT
ShortT
LongN
ShortN
c
Tumor vs. Nontumor
Canonical glycolysis
Total Protein
ALDOA
GAPDH
ALDOC
PFKP
PGM2L1
Heme biosynthesis
Oxygen transport
QDPR
UROD
CPOX
HBD
d
Tumor vs. Nontumor
Salivary and lung mucus protein
Total Protein
MUC5B
e
Short vs. Long (Tumor)
carbonyl reductase (NADPH) activity
haptoglobin binding
oxygen carrier activity
S100 protein binding
lipase inhibitor activity
cadherin binding in cell−cell adhesion
3−keto sterol reductase activity
phospholipase A2 inhibitor activity
peroxiredoxin activity
p.adjust
count
f
Short vs. Long (Tumor)
Calcium-binding protein S100P
Total Protein
S100P

Supplementary Fig. 4. Standard proteome analysis of LUAD cohort.

a, MS analysis shows consistent numbers of peptides and proteins detectable per each single sample pair of depleted (Dep) and total (Tot) extract. Solid line is the cumulative frequency number of unique proteins and/or protein groups over the experiment at an estimated false discovery rate (FDR) of 0,1% at peptide level. **b**, Heat map visualization of differentially expressed proteins. Manhattan distances used for hierarchical clustering. Differential expression analysis applied on proteins selected by PLS-DA (variate 1-2-3) and analysis adjusted for common confounders (i.e., age, gender, smoking status of participants). **c**, Differential expressions of proteins in tumor versus nontumor comparison: canonical glycolysis, oxygen transport, heme biosynthesis. **d**, Tumor expression of salivary gland specific protein Muc5B. **e**, GO enriched molecular function (MF) of proteins changed in survival-subtype tumor comparison. Functional enrichment GO analysis BH-adjusted P-value corresponds to two-sided Fisher's exact test. All genes in *Homo sapiens* database are used as reference list. **f**, Tumor expression of Calcium-binding protein S100P. Two-sided P-value from GLM (family = Gaussian), accounted for age, sex and smoking status (**c**, **d**, **f**). Post hoc analysis multiple comparison BH FDR-adjusted P-value ≤0.05 is considered significant. Color codes correspond to sample classes annotated in figure legend. Barplots with data points shown in (c, d, f) are mean values +/- SEM. Total protein on y-axis is log2 protein abundance. Source data are provided as a Source Data file.

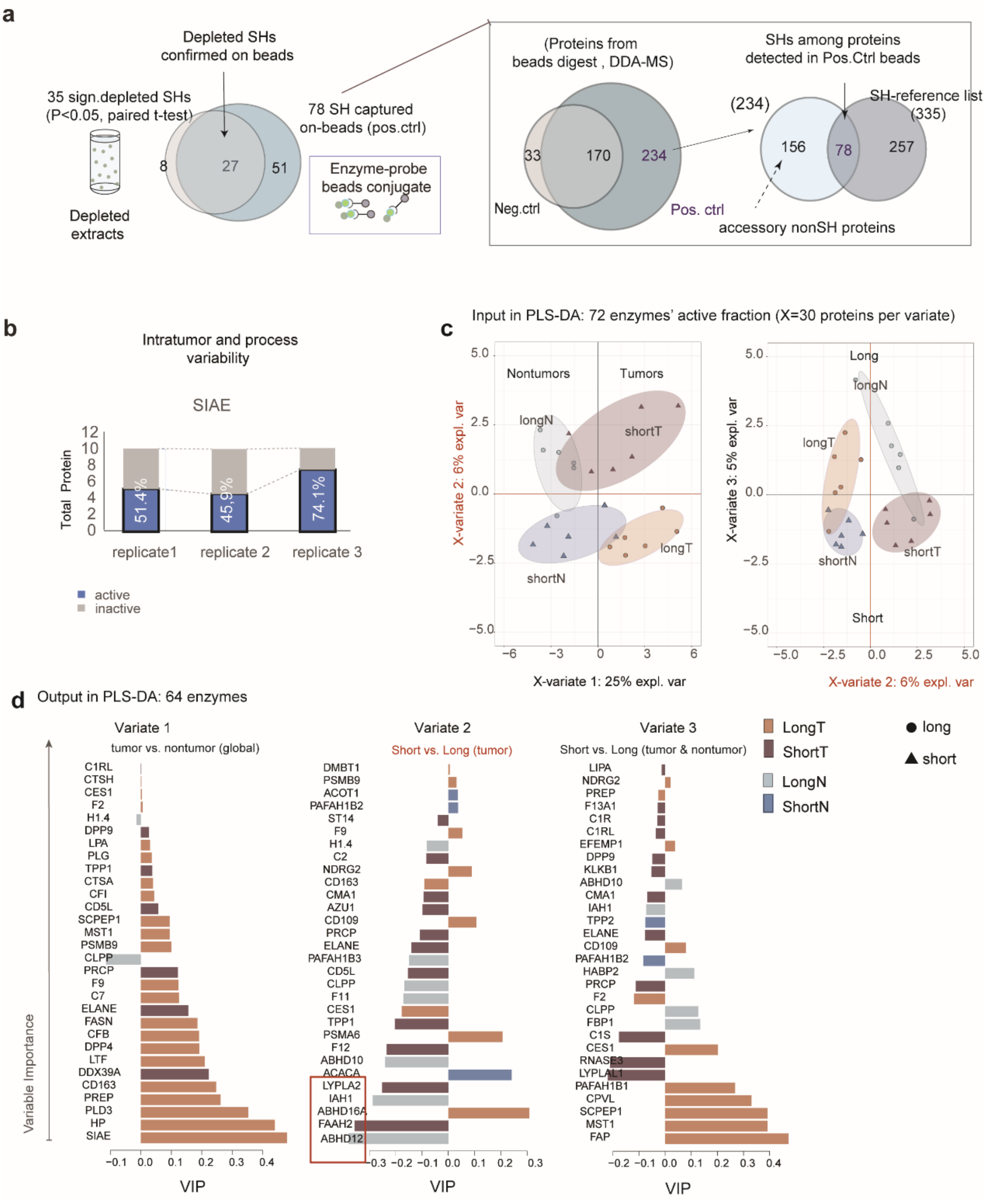
a
Depleted SHs confirmed on beads
35 sign.depleted SHs (P<0.05, paired t-test)
8
27
51
78 SH captured on-beads (pos.ctrl)
Depleted extracts
Enzyme-probe beads conjugate
(Proteins from beads digest , DDA-MS)
33
170
234
Neg.ctrl
Pos. ctrl
SHs among proteins detected in Pos.Ctrl beads
(234)
SH-reference list (335)
156
78
257
accessory nonSH proteins
b
Intratumor and process variability
SIAE
Total Protein
51.4%
45.9%
74.1%
replicate1
replicate 2
replicate 3
active
inactive
c
Input in PLS-DA: 72 enzymes' active fraction (X=30 proteins per variate)
Nontumors
Tumors
longN
shortT
shortN
longT
X-variate 2: 6% expl. var
X-variate 1: 25% expl. var
Long
Short
X-variate 3: 5% expl. var
d
Output in PLS-DA: 64 enzymes
Variate 1
tumor vs. nontumor (global)
Variate 2
Short vs. Long (tumor)
Variate 3
Short vs. Long (tumor & nontumor)
LongT
ShortT
LongN
ShortN
long
short
Variable Importance
VIP
C1RL CTSH CES1 F2 H1.4 DPP9 LPA PLG TPP1 CTSA CFI CD5L SCPEP1 MST1 PSMB9 CLPP PRCP F9 C7 ELANE FASN CFB DPP4 LTF DDX39A CD163 PREP PLD3 HP SIAE
DMBT1 PSMB9 ACOT1 PAFAH1B2 ST14 F9 H1.4 C2 NDRG2 CD163 CMA1 AZU1 CD109 PRCP ELANE PAFAH1B3 CD5L CLPP F11 CES1 TPP1 PSMA6 F12 ABHD10 ACACA LYPLA2 IAH1 ABHD16A FAAH2 ABHD12
LIPA NDRG2 PREP F13A1 C1R C1RL EFEMP1 DPP9 KLKB1 ABHD10 CMA1 IAH1 TPP2 ELANE CD109 PAFAH1B2 HABP2 PRCP F2 CLPP FBP1 C1S CES1 RNASE3 LYPLAL1 PAFAH1B1 CPVL SCPEP1 MST1 FAP

Supplementary Fig. 5. Percentage of active SH forms alter across LUAD groups.

a, Left panel: Venn diagram overlaps depict number of significantly depleted SHs (i.e., 27/35) confirmed with orthogonal beads experiment. Right panel: Venn diagrams from orthogonal experiment represent SHs enriched on avidin beads after incubation with FP-probe (Pos. ctrl) or DMSO reaction solvent (Neg. ctrl). Of 234 cases detected on Pos. ctrl, we confirm detection of 78 assigned SH enzymes. **b**, Degree of SIAE activity across three independent extracts of spatially separated tumor tissue cuts. **c**, Supervised PLS-DA classification analysis based on active SH fractions. Class separation based on latent variate 1 and 2, and latent variate 2 and 3. Limited number of features per component (N=30). **d**, Features contribution via VIP score to PLS-DA latent variate 1, 2 and 3. Color codes correspond to sample classes annotated in legend.

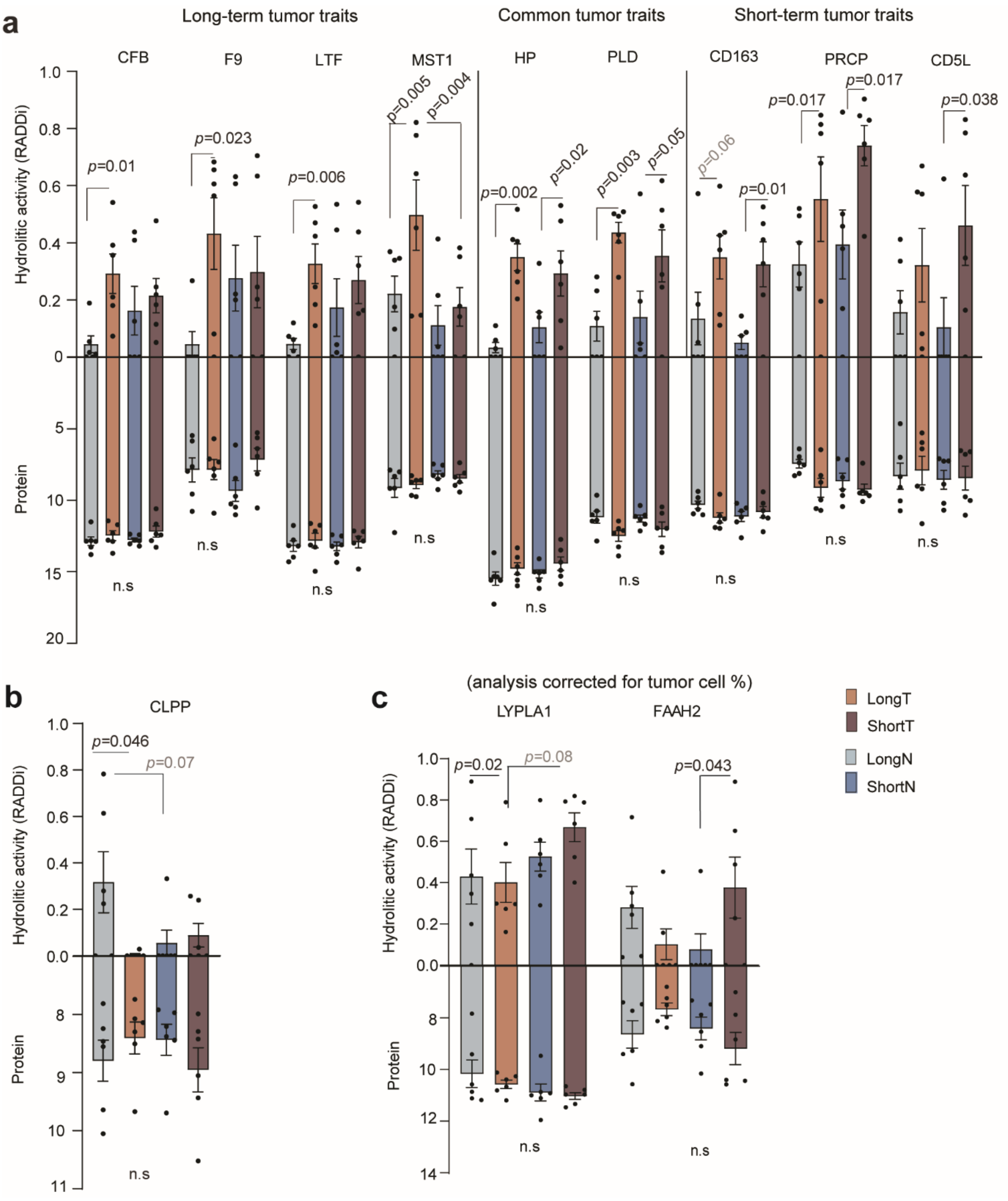
a
Long-term tumor traits
Common tumor traits
Short-term tumor traits
CFB
F9
LTF
MST1
HP
PLD
CD163
PRCP
CD5L
Hydrolitic activity (RADDi)
Protein
p=0.01
p=0.023
p=0.006
p=0.005
p=0.004
p=0.002
p=0.02
p=0.003
p=0.05
p=0.06
p=0.01
p=0.017
p=0.017
p=0.038
n.s
b
CLPP
p=0.046
p=0.07
c
(analysis corrected for tumor cell %)
LYPLA1
FAAH2
p=0.02
p=0.08
p=0.043
LongT
ShortT
LongN
ShortN

Supplementary Fig. 6. Profiling of SH activity in LUAD clinical cohort.

a-b, Barplots represent tumors' total and active fractions of dysregulated SHs. **a**, SHs that altered percentage of active fraction in tumors compared with adjacent nontumor tissues with LUAD survival subtype specificity. **b**, Barplot of CLPP active fractions. Two-sided P-value is the confounder-adjusted GLM (quasibinomial model) for age, gender, and smoking status of patients (**a-b**). **c**, Barplots of novel hits LYPLA1 and FAAH2 after analysis adjustement for percentage of tumor cells. Two-sided P-value of novel hits corresponds to GLM analysis (quasibinomial model) accounting for percentage of tumor cells estimated per tumor tissue cut (Supplementary Table 1). Barplots with data points (n=6 samples/per group) shown in (**a**, **b**, **c**) are mean values +/- SEM. Color codes correspond to conditions annotated in figure legend. Source data are provided as a Source Data file.

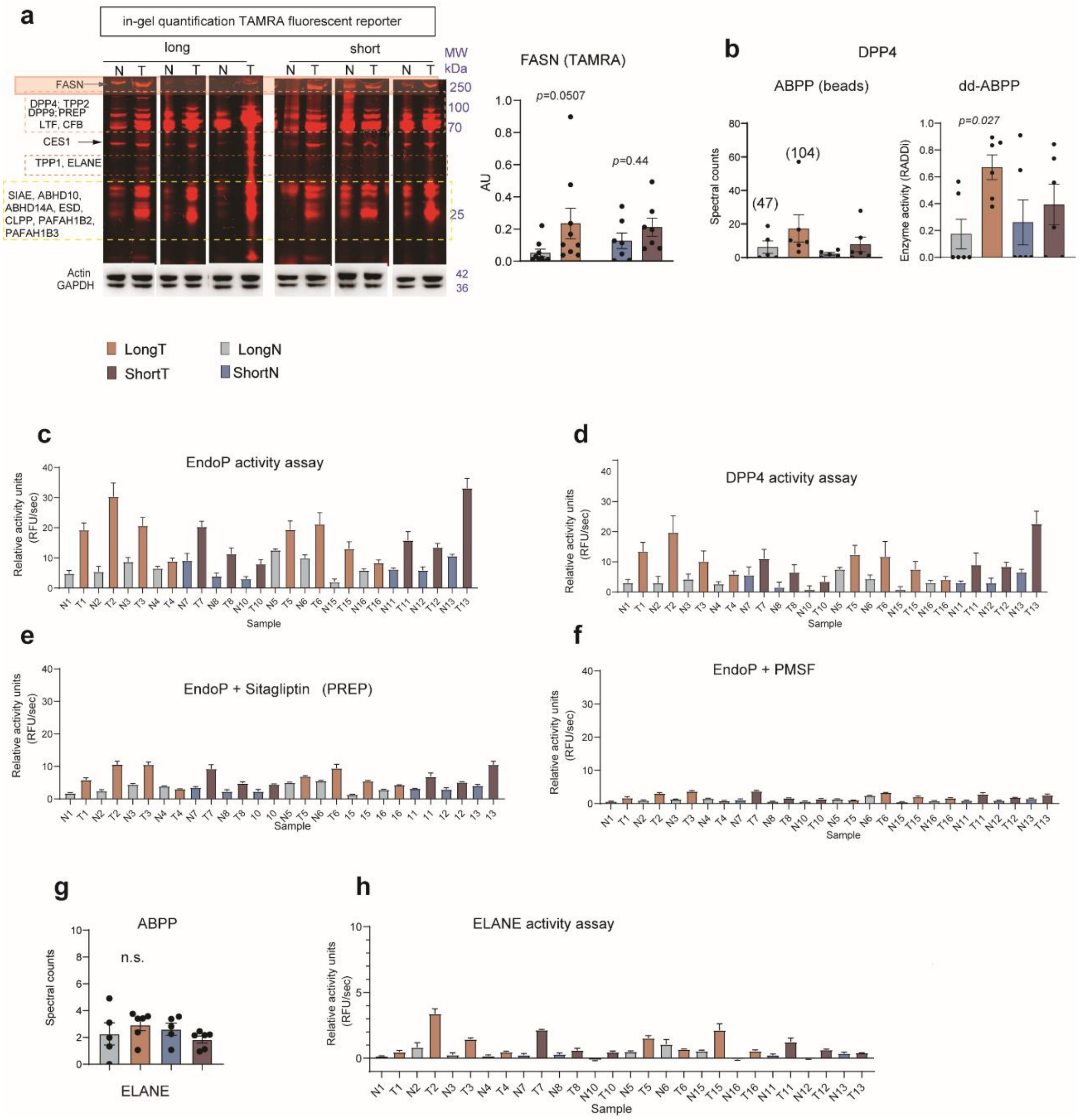
a
in-gel quantification TAMRA fluorescent reporter
long
short
N T N T N T N T N T N T
MW kDa
FASN
DPP4; TPP2 DPP9; PREP LTF, CFB
CES1
TPP1, ELANE
SIAE, ABHD10, ABHD14A, ESD, CLPP, PAFAH1B2, PAFAH1B3
250
100
70
25
Actin GAPDH
42
36
FASN (TAMRA)
p=0.0507
p=0.44
AU
b
DPP4
ABPP (beads)
dd-ABPP
(47)
(104)
Spectral counts
p=0.027
Enzyme activity (RADDi)
LongT
LongN
ShortT
ShortN
c
EndoP activity assay
Relative activity units (RFU/sec)
Sample
d
DPP4 activity assay
Relative activity units (RFU/sec)
Sample
e
EndoP + Sitagliptin (PREP)
Relative activity units (RFU/sec)
Sample
f
EndoP + PMSF
Relative activity units (RFU/sec)
Sample
g
ABPP
n.s.
Spectral counts
ELANE
h
ELANE activity assay
Relative activity units (RFU/sec)
Sample

Supplementary Fig. 7. Validation experiments by orthogonal methods.

a, Fluorescent image shows labeled gel-separated proteome and corresponding SHs detected by SC with in-gel proteomic digestion. SHs detected in-gel proteomic workflow annotated at side of image. Barplots of FASN represent activity status estimated via in-gel quantification and normalization to actin loading control. Two-sided p-value from Kruskal–Wallis test followed by Dunn's multiple comparisons adjustment. **b**, Barplots of DPP4 represent activity status of enzymes across LUAD groups estimated by novel dd-ABPP (n=6 per/group) and conventional ABPP via SC averaging (n=5 for LongN and ShortN; n=6 for LongT and ShortT). Barplots are presented as mean values +/- SEM. **c**, Barplots of individual samples corresponding to EndoP activity evaluated by functional assays. **d**, Barplots of individual samples corresponding to DPP4 activity evaluated by functional assays. **e**, Barplots of individual samples corresponding to residual activity after sitagliptin inhibition, reflecting other prolyl endopeptidases than DPP4, mostly PREP. Of note, PREP was among the strongest hits in dd-ABPP screen. **f**, PMSF inhibition test to confirm specificity of detected signals and confirm their attribution to serine protease family. **g**, The SC data of ELANE from LUAD conditions in ABPP analysis. Barplots are presented as mean values +/- SEM. (n=5 for LongN and ShortN; n=6 for LongT and ShortT) **h**, Individual sample records from ELANE activity assay. Color codes correspond to conditions annotated in figure legend. Source data are provided as a Source Data file.

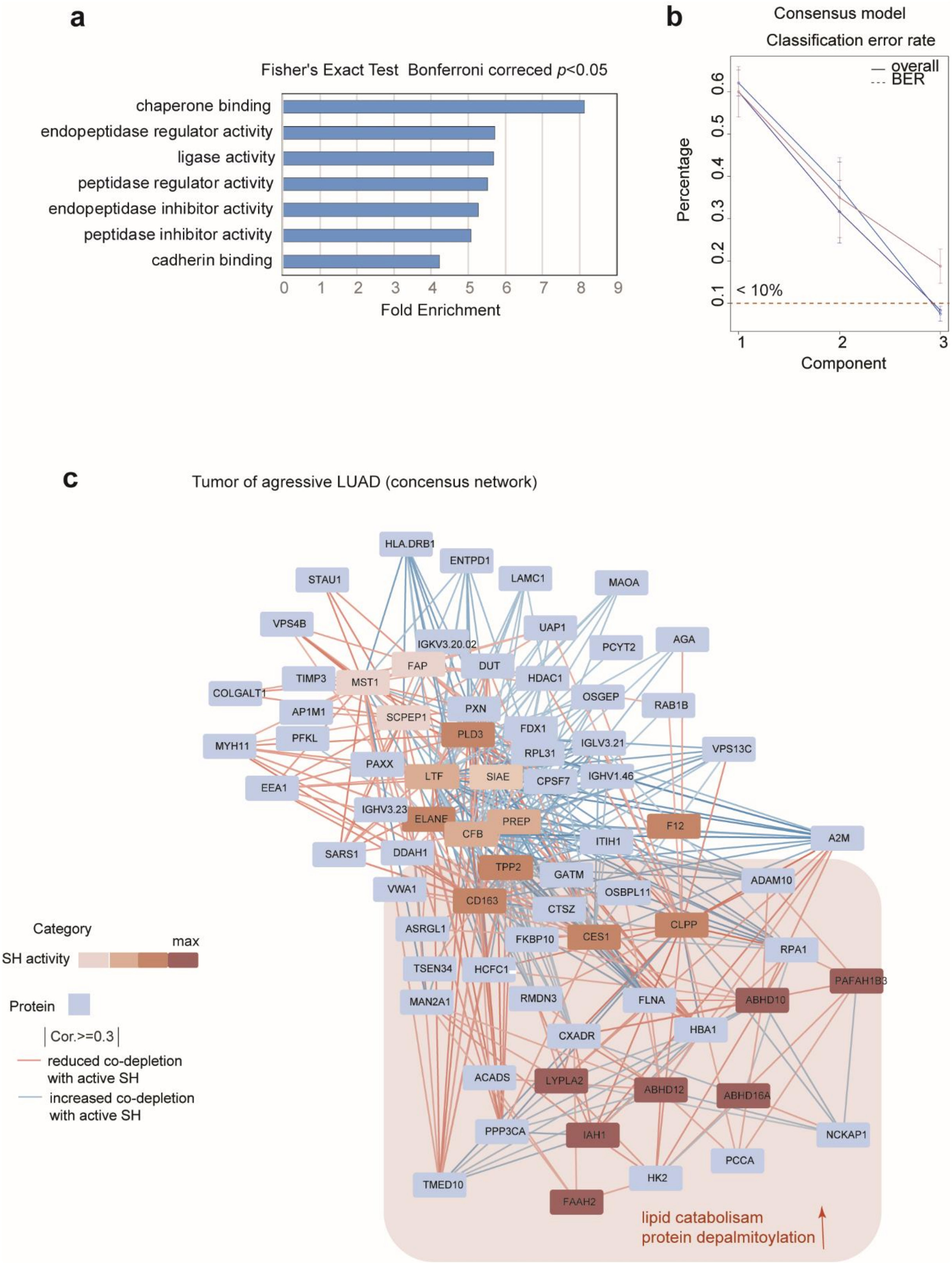
a
Fisher's Exact Test Bonferroni correced p<0.05
chaperone binding
endopeptidase regulator activity
ligase activity
peptidase regulator activity
endopeptidase inhibitor activity
peptidase inhibitor activity
cadherin binding
Fold Enrichment
b
Consensus model
Classification error rate
overall
BER
Percentage
< 10%
Component
c
Tumor of agressive LUAD (concensus network)
Category
SH activity
max
Protein
Cor.>=0.3
reduced co-depletion with active SH
increased co-depletion with active SH
lipid catabolisam
protein depalmitoylation

Supplementary Fig. 8. Comprehensive interaction networks of catalytic enzymes discriminate LUAD survival subtypes.

a, PANTHER Overrepresentation Test for GO analysis of molecular function conducted on 258 co-depleted proteins 1st degree SH interactors. Bonferroni adjusted *p*-value<0.05 corresponds to two-sided Fisher's exact test. All genes in Homo sapiens database are used as reference list. **b**, Consensus model performance error rate estimated by 5-fold cross-validation schema. Model selected via block sPLS-DA analysis conducted on 64 SHs and 388 co-depleted proteins. Block PLS-DA selects variables based on its i) maximum covariance between integrated data levels, and ii) ability to discriminate between sample classes. **c**, Network of selected SHs and proteins extracted from consensus model (82 features) that correlates above selected cor. cut-off (i.e., |cor >0.3|). Proteins and respective enzymes cluster according to functional properties. Source data are provided as a Source Data file.

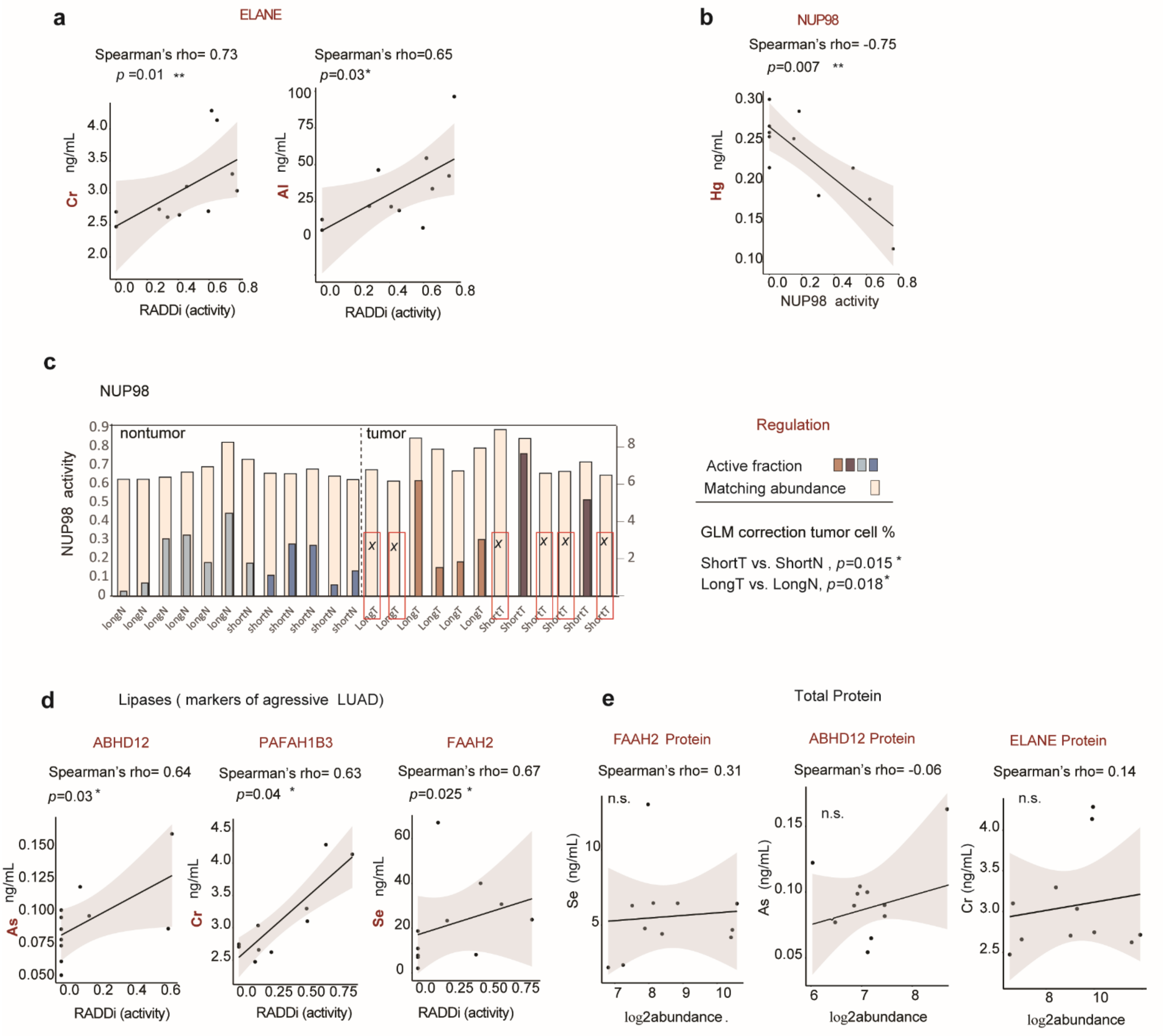

f

The top SHs with non-synonymus mutation in KRAS LUAD TCGA

Protein name	Number of patients with a non-synonimous mutation	Frequency of mutations (number of mutations divided with a gene length)	%patients with non-synonimus SH mutation in LUAD	Number of mes the patient with the mutation also had a KRAS mutation	Number of patients that also had a KRAS mutation divided by all patients with a mutation	Fraction of patients with non-synonimus SH mutation in KRAS LUAD
DMBT1	15.00	0.01	0.07	7.00	0.47	0.097
LPA	16.00	0.00	0.07	7.00	0.44	0.097
PCSK5	6.00	0.00	0.03	5.00	0.83	0.069
DPP10	8.00	0.01	0.03	5.00	0.62	0.069
NUP98	8.00	0.00	0.03	4.00	0.50	0.056
F9	9.00	0.02	0.04	4.00	0.44	0.056
PNLIPRP1	6.00	0.01	0.03	3.00	0.50	0.042

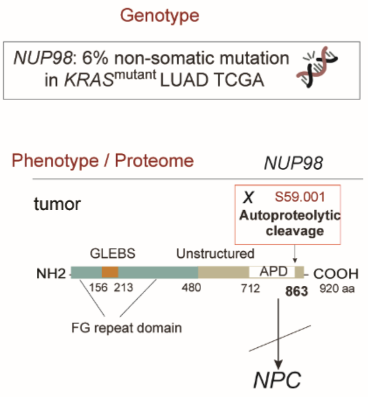

Supplementary Fig. 9. Tumor heterogeneity interferes with SH enzyme catalysis. a, Two scatter plots with 95% confidence intervals represent Spearman rho correlations of tumor contents of Cr and Al ions (ng/mL), and the active protease fraction of ELANE, respectively. **b**, Scatter plot of NUP98 activity correlation with Hg ion tumor content. **c**, Barplots with sample color code represent an active fraction of NUP98 estimated per individual sample. Red square depicts samples with missing NUP98 active fraction. Background barplots in light pink color represent protein expression of NUP98 per individual sample. Two-sided P-value from GLM adjusted for cell-count percentage per sample (see Supplementary Table 1) **d**, Scatter plots with 95% confidence intervals represent Spearman rho correlations of tumor contents of As, Cr, Se ions (ng/mL), and the active SHs' fraction of ABHD12, PAFAHB1B3, FAAH2, respectively. **e**, Representative scatter plots with Spearman correlation of measured total protein content of FAAH2, ABHD12 and ELANE along with lung concentration for Se, As, and Cr. **f**, Table of top serine hydrolases that show higher rate (>4%) of non-synonymous gene mutation across KRAS LUAD TCGA atlas. Percentage depicts fraction of patients with non-synonymous NUP98 mutations in KRAS mutated LUAD (TCGA). On the right: Domain architecture of human Nup98 protein of 98 kDa. Lack of autoproteolytic cleavage at amino acid position 863 fails to remove a C-terminal fragment of 8 kDa and target protein to the Nuclear Pore Complex. APD is an autoproteolytic S59 endopeptidase protein domain. Source data are provided as a Source Data file.

Supplementary Table 1. (a) Differential GLM (family =quasibinominal) analysis results for the percentage of active SH fractions across LUAD conditions accounting for confounders such as age, gender and smoking status of patients. P-values are two-sided, with no adjustments made for multiple comparaisons. Optional results from non-adjusted and cell-count adjuste differential analysis in (b) and (c), respectivly.

(a) P-value from GLM (family=quasibinomial) analysis accounting for age, gender and smoking status. Age represents subject's age at diagnosis. P values are two-sided, with no adjustments made for multiple comparisons. The effect size or standardizing coefficients in the brackets of relevant hits.				(b) P-value from GLM (family=quasibinomial) analysis and non-adjusted for confounders. P values are two-sided, with no adjustments made for multiple comparisons. The effect size or standardizing coefficients in the brackets of relevant hits.				(c) P-value from GLM (family=quasibinomial) accounting for age, gender, smoking, and % of tumor cells per tissue cut. P values are two-sided, with no adjustments made for multiple comparisons. Novel hits in bold.			
Tumor short survival vs. tumor long survival	Short survival tumor vs. short survival nontumor	Long survival tumor vs. long survival nontumor	Nontumor short survival vs. nontumor long survival	Tumor short survival vs. tumor long survival	Short survival tumor vs. short survival nontumor	Long survival tumor vs. long survival nontumor	Nontumor short survival vs. nontumor long survival	Tumor short survival vs. tumor long survival	Short survival tumor vs. short survival nontumor	Long survival tumor vs. long survival nontumor	Nontumor short survival vs. nontumor long survival
ABHD10: 0.2531	ABHD10: 0.0931	ABHD10: 0.1122	ABHD10: 0.047 (-1.40)	ABHD10: 0.3941	ABHD10: 0.1212	ABHD10: 0.1574	ABHD10: 0.05 (-1.39)	ABHD10: 0.3549	ABHD10: 0.1467	ABHD10: 0.4813	ABHD10: 0.05 (-1.4)
ABHD12: 0.1008	ABHD12: 0.1146	ABHD12: 0.032 (-1.80)	ABHD12: 0.025 (-1.43)	ABHD12: 0.0711	ABHD12: 0.0974	ABHD12: 0.027 (-1.67)	ABHD12: 0.03 (-1.30)	ABHD12: 0.1521	ABHD12: 0.0878	ABHD12: 0.2365	ABHD12: 0.027 (-1.42)
ABHD16A: 0.1333	ABHD16A: 0.1831	ABHD16A: 0.0667	ABHD16A: 0.0940	ABHD16A: 0.2112	ABHD16A: 0.1756	ABHD16A: 0.0726	ABHD16A: 0.0589	ABHD16A: 0.0739	ABHD16A: 0.9610	ABHD16A: 0.04 (2.54)	ABHD16A: 0.1046
C1S: 0.2903	C1S: 0.5609	C1S: 0.0607	C1S: 0.031 (1.57)	C1S: 0.7278	C1S: 0.6926	C1S: 0.1317	C1S: 0.1421	C1S: 0.2043	C1S: 0.9081	C1S: 0.4635	C1S: 0.032 (1.60)
CD163: 0.6948	CD163: 0.012 (1.187)	CD163: 0.0574	CD163: 0.2463	CD163: 0.8433	CD163: 0.01 (1.18)	CD163: 0.06	CD163: 0.2766	CD163: 0.3349	CD163: 0.0045 (1.63)	CD163: 0.027 (1.44)	CD163: 0.2548
CD5L: 0.4470	CD5L: 0.038 (1.26)	CD5L: 0.3096	CD5L: 0.6878	CD5L: 0.4713	CD5L: 0.049 (1.16)	CD5L: 0.3316	CD5L: 0.6916	CD5L: 0.2536	CD5L: 0.3378	CD5L: 0.8206	CD5L: 0.6773
CES1: 0.8366	CES1: 0.035 (0.77)	CES1: 0.4993	CES1: 0.1016	CES1: 0.8404	CES1: 0.031 (0.76)	CES1: 0.4885	CES1: 0.0910	CES1: 0.7282	CES1: 0.0798	CES1: 0.4842	CES1: 0.1121
CFB: 0.4081	CFB: 0.5826	CFB: 0.01 (1.18)	CFB: 0.1559	CFB: 0.4685	CFB: 0.5786	CFB: 0.009 (1.14)	CFB: 0.1196	CFB: 0.2839	CFB: 0.3599	CFB: 0.0248 (1.58)	CFB: 0.1594
CLPP: 0.1691	CLPP: 0.6697	CLPP: 0.047 (-2.4)	CLPP: 0.0791	CLPP: 0.2597	CLPP: 0.6632	CLPP: 0.049 (-2.11)	CLPP: 0.0275 (0.29)	CLPP: 0.1534	CLPP: 0.9226	CLPP: 0.0718	CLPP: 0.0859
DPP4: 0.1860	DPP4: 0.5264	DPP4: 0.027 (1.40)	DPP4: 0.7125	DPP4: 0.1928	DPP4: 0.5065	DPP4: 0.02 (1.38)	DPP4: 0.6230	DPP4: 0.0835	DPP4: 0.1570	DPP4: 0.0176 (2.60)	DPP4: 0.7054
ELANE: 0.4462	ELANE: 0.0129 (1.06)	ELANE: 0.0751	ELANE: 0.9158	ELANE: 0.2915	ELANE: 0.024 (1.02)	ELANE: 0.1260	ELANE: 0.7693	ELANE: 0.3074	ELANE: 0.2183	ELANE: 0.6836	ELANE: 0.9317
F9: 0.5067	F9: 0.8896	F9: 0.023 (1.54)	F9: 0.1016	F9: 0.4325	F9: 0.8939	F9: 0.016 (1.52)	F9: 0.0858	F9: 0.3268	F9: 0.4487	F9: 0.0286 (2.25)	F9: 0.1043
FAAH2: 0.1232	FAAH2: 0.0758	FAAH2: 0.2370	FAAH2: 0.1607	FAAH2: 0.0939	FAAH2: 0.0645	FAAH2: 0.2336	FAAH2: 0.1648	FAAH2: 0.3564	FAAH2: 0.043 (1.70)	FAAH2: 0.8710	FAAH2: 0.1742
FASN: 0.5638	FASN: 0.3445	FASN: 0.018 (1.03)	FASN: 0.3077	FASN: 0.7152	FASN: 0.3486	FASN: 0.021 (0.99)	FASN: 0.2543	FASN: 0.3083	FASN: 0.1453	FASN: 0.0179 (1.62)	FASN: 0.3038
HP: 0.3982	HP: 0.0214 (0.71)	HP: 0.0002 (0.71)	HP: 0.2098	HP: 0.5433	HP: 0.02 (0.77)	HP: 0.0002 (1.45)	HP: 0.1694	HP: 0.1600	HP: 0.006 (1.07)	HP: 0.0002 (2.05)	HP: 0.1996
IAH1: 0.0179 (1:79)	IAH1: 0.3299	IAH1: 0.016 (1.82)	IAH1: 0.3132	IAH1: 0.0134 (1.77)	IAH1: 0.3337	IAH1: 0.014 (-1.76)	IAH1: 0.3417	IAH1: 0.0076 (2.15)	IAH1: 0.7547	IAH1: 0.008 (-2.8)	IAH1: 0.3018
LTF: 0.5693	LTF: 0.3501	LTF: 0.006 (f2: 2.03)	LTF: 0.1251	LTF: 0.6318	LTF: 0.3745	LTF: 0.008 (1.23)	LTF: 0.1241	LTF: 0.2695	LTF: 0.1231	LTF: 0.0054 (1.97)	LTF: 0.1200
LYPLA1: 0.3636	LYPLA1: 0.9632	LYPLA1: 0.2533	LYPLA1: 0.7811	LYPLA1: 0.4354	LYPLA1: 0.9575	LYPLA1: 0.2567	LYPLA1: 0.6795	LYPLA1: 0.0885	LYPLA1: 0.1726	LYPLA1: 0.021 (-1.85)	LYPLA1: 0.7455
LYPLA2: 0.044 (1.03)	LYPLA2: 0.021 (1.35)	LYPLA2: 0.1416	LYPLA2: 0.0785	LYPLA2: 0.1571	LYPLA2: 0.0808	LYPLA2: 0.2258	LYPLA2: 0.1185	LYPLA2: 0.0762	LYPLA2: 0.0768	LYPLA2: 0.3819	LYPLA2: 0.0884
LYPLAL1: 0.0245 (0.71)	LYPLAL1: 0.2204	LYPLAL1: 0.8079	LYPLAL1: 0.4048	LYPLAL1: 0.053 (0.68)	LYPLAL1: 0.2954	LYPLAL1: 0.8325	LYPLAL1: 0.4846	LYPLAL1: 0.1108	LYPLAL1: 0.1218	LYPLAL1: 0.4998	LYPLAL1: 0.3976
MST1: 0.0044 (-0.92)	MST1: 0.3409	MST1: 0.005 (0.88)	MST1: 0.2846	MST1: 0.018 (-0.92)	MST1: 0.5197	MST1: 0.045 (0.76)	MST1: 0.3018	MST1: 0.0975	MST1: 0.3451	MST1: 0.5433	MST1: 0.2082
PAFAH1B2: 0.9347	PAFAH1B2: 0.3268	PAFAH1B2: 0.9505	PAFAH1B2: 0.3288	PAFAH1B2: 0.9499	PAFAH1B2: 0.2911	PAFAH1B2: 0.9678	PAFAH1B2: 0.2814	PAFAH1B2: 0.4089	PAFAH1B2: 0.03 (-2.53)	PAFAH1B2: 0.037 (-2.56)	PAFAH1B2: 0.2645
PLD3: 0.3841	PLD3: 0.05	PLD3: 0.0027 (1.14)	PLD3: 0.7356	PLD3: 0.5254	PLD3: 0.0606	PLD3: 0.006 (1.03)	PLD3: 0.7121	PLD3: 0.1606	PLD3: 0.017 (1.11)	PLD3: 0.0036 (1.79)	PLD3: 0.7261
PRCP: 0.3262	PRCP: 0.0179 (0.97)	PRCP: 0.1207	PRCP: 0.8875	PRCP: 0.2203	PRCP: 0.029 (0.59)	PRCP: 0.1491	PRCP: 0.6472	PRCP: 0.6613	PRCP: 0.0196 (1.45)	PRCP: 0.0809	PRCP: 0.8682
PREP: 0.8536	PREP: 0.2229	PREP: 0.018 (1.04)	PREP: 0.2911	PREP: 0.8626	PREP: 0.1848	PREP: 0.0096 (1.04)	PREP: 0.2277	PREP: 0.9956	PREP: 0.5463	PREP: 0.2819	PREP: 0.2996
SCPEP1: 0.004 (-0.92)	SCPEP1: 0.3409	SCPEP1: 0.005 (0.88)	SCPEP1: 0.2846	SCPEP1: 0.018 (-0.92)	SCPEP1: 0.5197	SCPEP1: 0.045 (0.76)	SCPEP1: 0.3018	SCPEP1: 0.0975	SCPEP1: 0.3451	SCPEP1: 0.5433	SCPEP1: 0.2082
SIAE: 0.1796	SIAE: 0.0261 (0.70)	SIAE: 0.0000 (1.48)	SIAE: 0.2950	SIAE: 0.1004	SIAE: 0.0305 (0.67)	SIAE: 0.0000 (1.43)	SIAE: 0.4392	SIAE: 0.0435 (-0.66)	SIAE: 0.0033 (1.22)	SIAE: 0.0000 (2.26)	SIAE: 0.2714
TPP1: 0.3718	TPP1: 0.039 (1.20)	TPP1: 0.3795	TPP1: 0.6286	TPP1: 0.4383	TPP1: 0.0342 (1.16)	TPP1: 0.3929	TPP1: 0.5051	TPP1: 0.7874	TPP1: 0.0247 (1.63)	TPP1: 0.1835	TPP1: 0.6393
NUP98: 0.6703	NUP98: 0.7988	NUP98: 0.9978	NUP98: 0.8635	NUP98: 0.9778	NUP98: 0.7467	NUP98: 0.9870	NUP98: 0.7554	NUP98: 0.1030	NUP98: 0.0153 (2.97)	NUP98: 0.0185 (1.19)	NUP98: 0.6055

Supplementary Notes

Supplementary Note 1: Original images for Western blots and gels Figure 4a and Figure 6c, respectively.

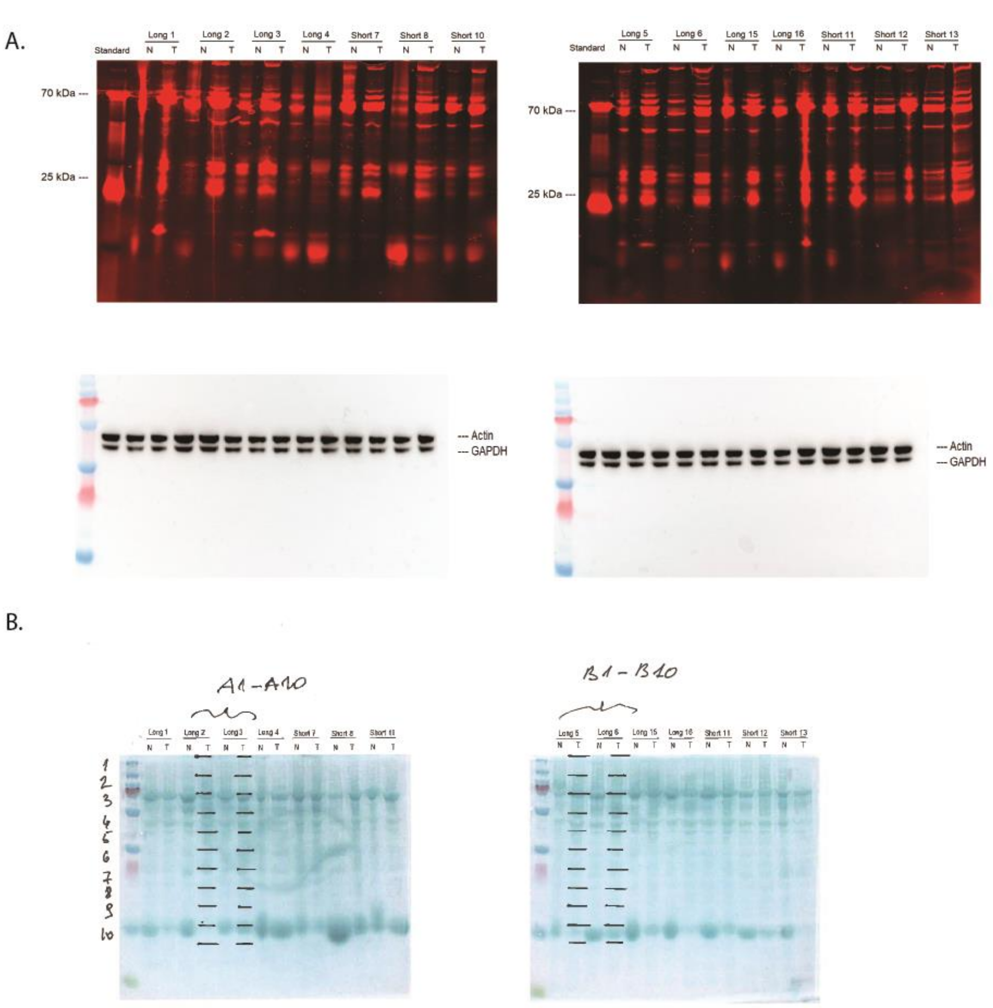

Figure 4a. (A). Upper: Fluorescent gels – original images generated after sample separation by gel electrophoresis. Protein extracts tagged with TAMRA fluorescence reporter. Bottom: Western blot with actin and GAPDH loading control. ImageJ software was used to quantify the bands. Results reported in Fig. 4D-E. (B). Coomassie brilliant blue staining of gels. Representative samples selected per gel and the respective protein lanes cut into 10 slices (gel on the left: A1-A10; gel on the right: B1-B10) following a standard in-gel protocol for identifications of proteins per bands appearing on the gel.

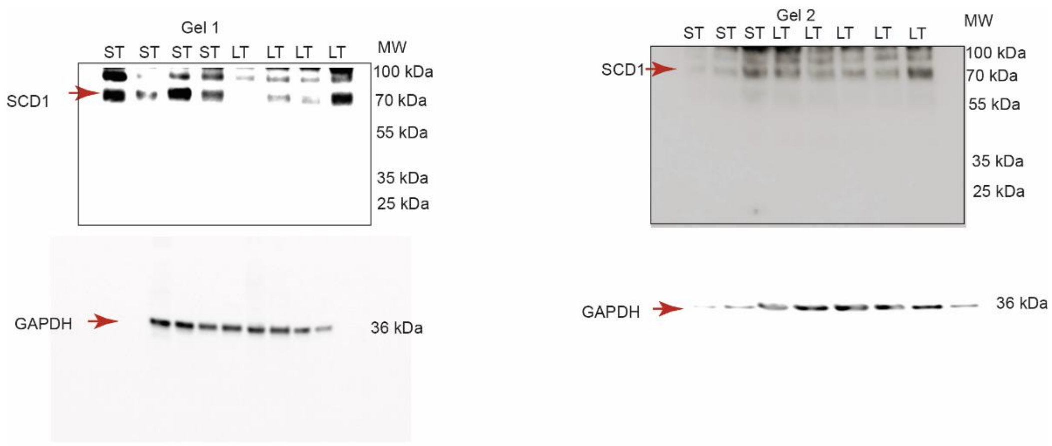

Figure 6b. Original images for SCD1 western blot. Upper: Membranes of respective gel1 (left) and gel 2 (right) – original images generated after sample separation by gel electrophoresis. Protein extracts of tumor tissues from Long survival patients (LT) and from Short-suvival patients (ST). Bottom: Western blot with GAPDH loading control for each respective sample. ImageJ software was used to quantify the bands. Results reported in Fig. 6.c. SCD1 molecular weight (MW) predicted at 42 kDa, and detected at 70 kDa.

Supplementary Note 2: Overview of the characteristics of each proteomics method for studying enzyme families, the new generation of dd-ABPP and the standard ABPP approach.

Method	Description of procedure		Quantitative data reports	Method advantages
**dd-ABPP SWATH/DIA-MS** 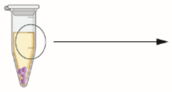	Data acquisition	• DIA-MS with spectral library	• Fraction of active enzymes<br>• Abundance of enzymes<br>• Abundance of proteins (contextual proteome)<br>• Protein interactors co-depleted with active enzymes	• Streamlined *in-solution* protein digestion<br>• No streptavidin contamination<br>• No time-consuming beads washing<br>• Total and active form of enzymes of interest available<br>• Contextual sample proteome and protein interactors with enzymes available
	Protein digestion	• In-solution digestion		
	Analysed samples	• Total tissue extract<br>• Depleted tissue extract for desactivated enzymes		
**ABPP-MS** 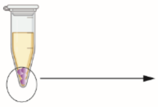	Data acquisition	• DDA-MS	• Active enzymes<br>• Accesory non-enzyme proteins (potential interactors)	• Detection of probe-inactivated enzymes that allows specific discovery of new members
	Protein digestion	• On-bead digestion		
	Analysed samples	• Streptavidin beads pull-down enriched for desactivated enzymes<br>• Streptavidin beads pull-down with contaminants		

Supplementary Note 3: Relative comparison of selected FA in patient tumors obtained through a full scan untargeted LC-HRMS profiling on Agilent 6550 IonFunnel QTOF operating in negative ESI mode. FA annotation was done by the accurate mass and retention time (AMRT) matching against in-house database of pure lipid standards analyzed under the same analytical condition.

Table S. Note3: List of commercial lipid standards obtained from Sigma-Aldrich (Darmstadt, Germany) analysed as mixture of saturated, monounsaturated and unsaturated FA under the same analytical condition as tumour samples (UHPLC-HRMS Agilent QTOF system).

Saturated Fatty acids					
**Synonym**	**Name**	**Carbon/Ins**	**Sigma-Aldrich Ref**	[M-H]- (m/z)	**RT (min)**
Hexanoic	Caproic	C6:0	21529-5ML	**115.0765**	0.51
Octanoic	Caprylic	C8:0	C2875	**143.1078**	0.68
Decanoic	Capric	C10:0	C1875	**171.1391**	1.03
Dodecanoic	Lauric	C12:0	W261408	**199.1704**	1.67
Tetradecanoic	Myristic	C14:0	70082	**227.2017**	2.65
Hexadecanoic	Palmitic	C16:0	P0500	**255.2330**	3.37
Heptadecanoic	Margaric	C17:0	H3500	**269.2486**	3.71
Octadenoic	Stearic	C18:0	S4751	**283.2643**	4.08
Eicosanoic	Arachidic	C20:0	A3631	**311.2956**	4.9
Docosanoic	Behenic	C22:0	216941	**339.3269**	5.8
Tetracosanoic	Lignoceric	C24:0	L6641	**367.3582**	6.8
Hexacosanoic acid	Cerotic	C26:0	H0388	**395.3895**	7.8
Monoenoic fatty acids					
**Synonym**	**Name**	**Carbon/Ins**	**Sigma-Aldrich Ref**	**M-H-**	**RT (min)**
cis-9-hexadecenoic	Palmitoleic	C16:1 (n-7)	P9417	**253.2173**	2.87
cis-6-octadecenoic	Petroselinic	C18:1 (n-6)	P8750	**281.2486**	3.58
cis-9-octadecenoic	Oleic	C18:1 (n-9)	O1008	**281.2486**	3.51
trans-9-octadeceno	Elaidic	C18:1 (z-9)	E4637	**281.2486**	3.61
cis-11-octadecenoic	cis-vaccenic	C18:1 (n-7)	V0384	**281.2486**	3.49
cis-13-docodecenoi	Erucic	C22:1 (n-9)	E3385	**337.3112**	4.99
cis-15-tetracosenoic	Nervonic	C24:1 (n-9)	N1514	**365.3425**	5.89
Polyunsaturated fatty acids					
**Synonym**	**Name**	**Carbon/Ins**	**Sigma-Aldrich Ref**	**M-H-**	**RT (min)**
9,12-octadecadieno	Linoleic	18:2 (n-6)	L1012	**279.2330**	3.08
6,9,12-octadecatrie	y-linolenic	18:3 (n-6)	62174-100MG-F	**277.2173**	2.67
9,12,15-octadecatri	a-linolenic	18:3 (n-3)	L2376	**277.2173**	2.59
5,8,11,14-eicosatetr	arachidonic	20:4 (n-6)	23401-50MG	**303.2330**	3.01
5,8,11,14-17-eicosa	Eicosapentaenoic	20:5 (n-3)	E2011	**301.2173**	2.49
4,7,10,13,16,19-doc	Docosahexaenoic	22:6 (n-3)	D2534	**327.2330**	2.87

Figure S. Note3.1: Overlay of Total ion current (TIC) chromatograms of the 10 tumor extracts measured on LC-HRMS Agilent 6550 IonFunnel QTOF system.

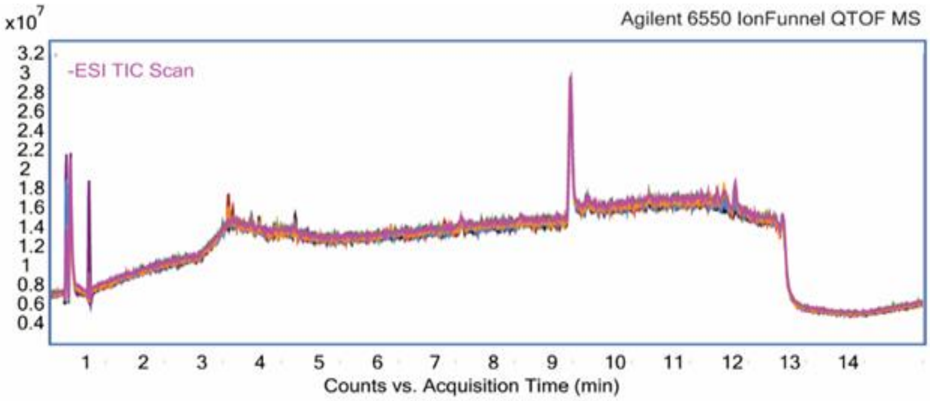

Figure S. Note3.2: Extracted ion chromatograms (EIC) of selected FAs annotated in the tumor extracts, with EICs of pure standards and blank (or neat solvent) analyzed under the same analytical conditions. The following chromatograms illustrate the saturated, monounsaturated (MUFA), and polyunsaturated (PUFA) FAs detected in the participants' tissue extracts based on AMRT matching to pure standards using MassHunter software (v:10.0; Agilent Technologies).

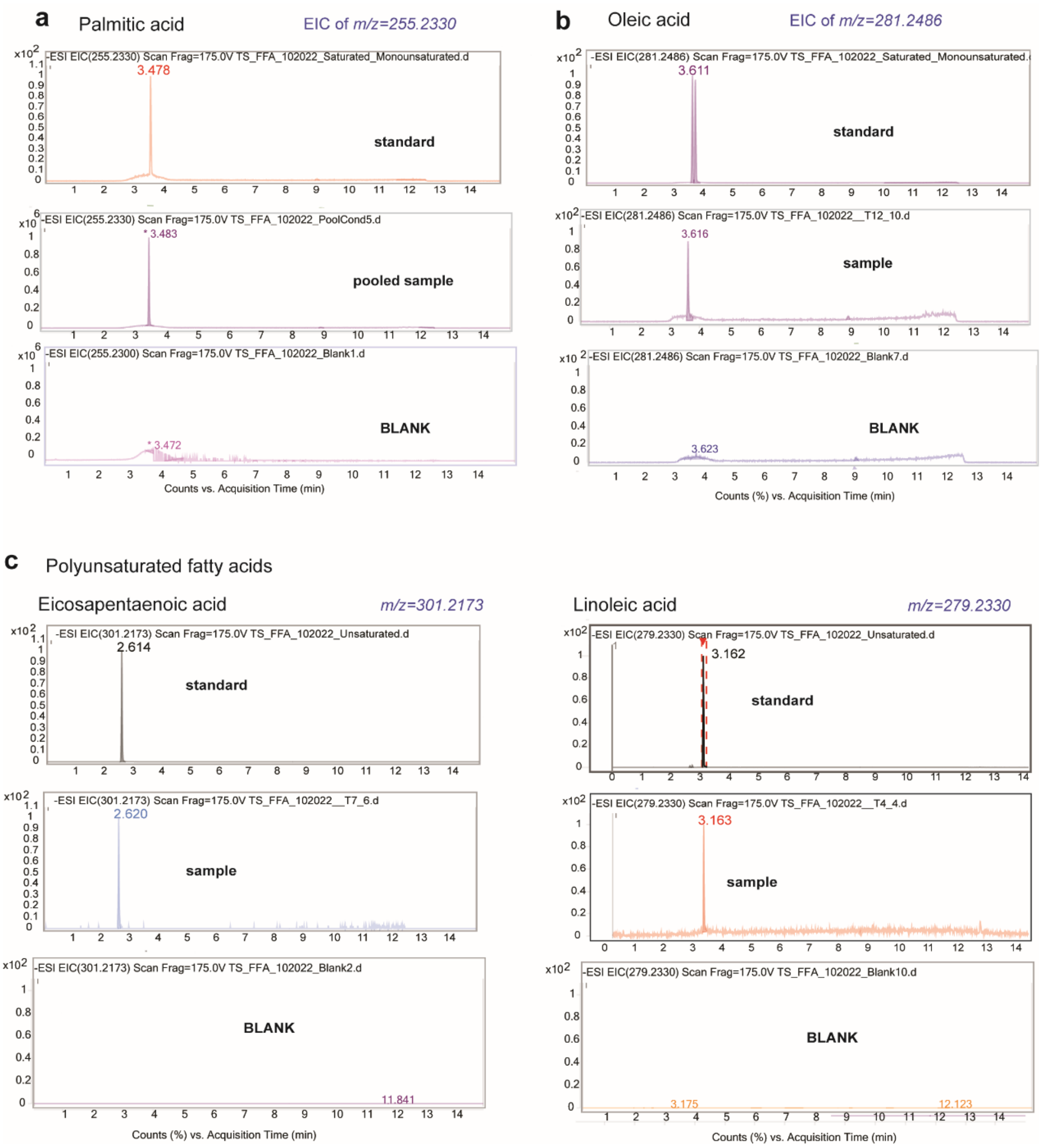

Supplementary Note 4: Absolute quantification of four FA of interest using UHPLC-HRMS Orbitrap™ IQ-X™ Tribrid™ mass spectrometer in ESI negative mode. For the absolute quantification of selected FA, external calibration curves based on the acquisition of pure authentic standards (AS) solutions against the isotopically labeled internal standard of known concentration were used.

Table S.Note4: List of commercial isotopically labelled internal standards obtained from Larodan AG (Solna, Sweden) used for absolute quantification of four FA of interest.

Reference Larodan AG (Solna, Sweden)	Molecular formula	Molecular weight	RT
Hexadecenoicacid-d3	C16H29D3O2	259.44	3.46
Palmitoleicacid-d13	C16H17D13O2	267.49	2.9
Oleicacid-d9	C18H25D9O2	291.52	3.58
Eicosapentaenoicacid-d5	C20H25D5O2	307.48	2.49

Figure S. Note4.**1** TIC sample chromatograms on LC-HRMS using Orbitrap IQX Tribrid MS **-** in "superimposed mode" show that fresh frozen tissue (middle panel, Tu6 was available as fresh frozen) has the same TIC profiles as OCT cleaned tissue.

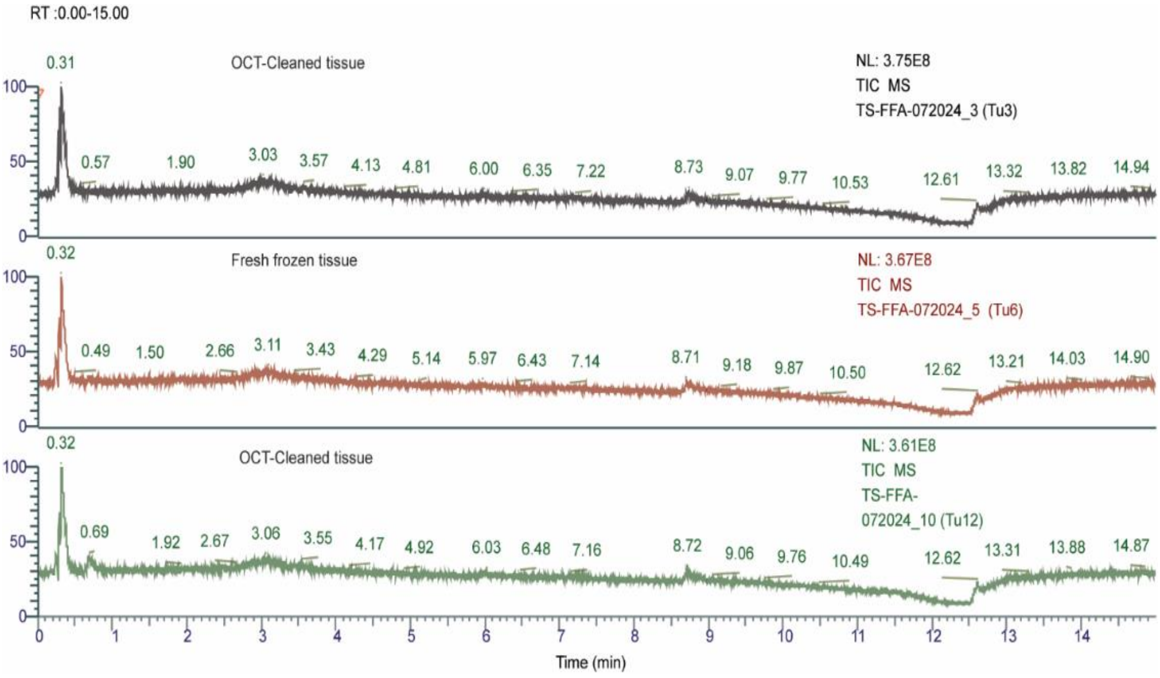

Figure S. Note4.2 Extracted ion chromatograms (EICs) for the measured fatty acids show the peak shape and RT in the: 1.) one tissue lysate (sample extract), the extracted blank or calibrator 0 (processed in the same way as samples) and 3) blank or neat solvent. EIC of their deuterated internal standards (IS) is on the first upper panel. Quantitative accuracy is ensured by the linearity of calibration curve, following the manual peak integration and background subtraction. The linearity of the external calibration curves was evaluated for each lipid using a 9-point range (Cal0-Cal9). Despite the peak shape, when integrated manually respective peak areas nicely align and show linear MS signal response. In addition, the internal standard (IS) behaves in the same way in the same analytical conditions which allowed for the matrix effect correction for improved quantification accuracy. Plots the EICs reported by FreeStyle™ Thermo Fisher Scientific (v. 1.8.65.0) software.

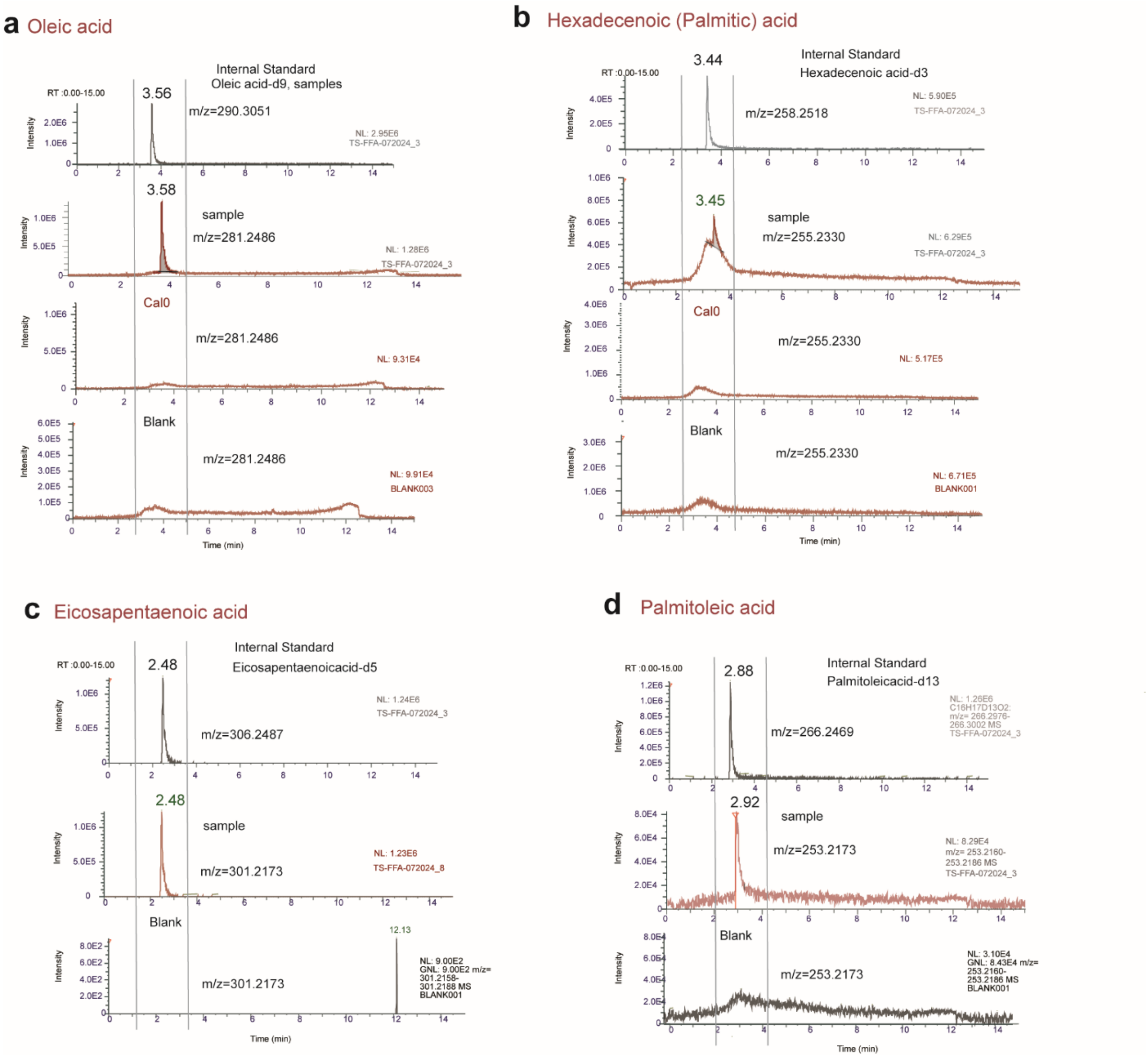

Supplementary Note 5. Minimal Reporting Checklist (https://lipidomicstandards.org/) for lipid measurements presented in the Figure **6** in two independent LC-MS platforms: A) relative quantification on Agilent QTOF system and B) absolute quantification on Orbitrap IQX Tribrid MS.

Created by https://lipidomicstandards.org, version v2.4.0

Contents of Report

Separation Workflow 1
Overall study design . . . 1
Lipid extraction . . . 1
Analytical platform . . . 1
Quality control . . . 2
Method qualification and validation . . . 2
Reporting . . . 2

Sample Descriptions 2
FullMSscan_FFA analysis / Human / Tissues (e.g., liver, heart, brain) . . . 2

Lipid Class Descriptions 3
1) FA[M-H]- / Lipid identification . . . 3
1) FA[M-H]- / Lipid quantification . . . 4

Separation Workflow

Overall study design

Title of the study	Depletion-dependent Activity-Based Protein Profiling coupled to SWATH/DIA Mass Spectrometry detects serine hydrolase lipid remodeling in lung adenocarcinoma progression		
Document creation date	02/10/2025	Corresponding Email	tatjana.sajic@chuv.ch
Principal investigator	Tatjana Sajic	Is the workflow targeted or untargeted?	Untargeted
Institution	Lausanne University Hospital	Clinical	Yes

Lipid extraction

Extraction method	2-phase system	Were internal standards added prior extraction?	No
pH adjustment	PBS	Special conditions	First PBS 1X; Isopropanol
2-phase system	2-step extraction	Derivatization	No

Analytical platform

Ionizaton additives	Ammonium acetate	Ion source	ESI
Number of separation dimensions	One dimension	MS Level	MS1
Separation type 1	LC	Mass resolution for detected ion at MS1	High resolution
Separation mode 1 (liquid)	RP	Resolution at m/z 200 at MS1	20000
Detector	Mass spectrometer	Mass accuracy in ppm at MS1	5
MS type	QTOF	Recording mode of raw data at MS1	Centroid mode
MS vendor	Agilent	Was/Were additional dimension/techniques used	No

Quality control

Blanks	Yes	Quality control	Yes
Type of Blanks	Extraction blank, Solvent blank	Type of QC sample	Commercial sample, Sample pool

Method qualification and validation

Method validation	Yes	Precision	No
Lipid recovery	Yes	Accuracy	Yes
Dynamic quantification range	Yes	Guidelines followed	EMA
Limit of quantitation (LOQ)/Limit of detection (LOD)	No		

Reporting

Are reported raw data uploaded into repository?	Yes	Summary data	Quantification and identification data
Link to repository / ID to entry	10.5281/zenodo.14841692	Raw data upload	Yes
Are metadata available?	Yes	Additional comments	Relative comparison of peak areas of FA

Sample Descriptions

FullMSscan_FFA analysis / Human / Tissues (e.g., liver, heart, brain)

Perfusion	No	Additives	None
Storage and collection conditions	Available	Were samples stored under inert gas?	No
Provided preanalytical information	-	Additional preservation methods	No
Temperature handling original sample	-20 °C	Biobank samples	Yes
Instant sample preparation	Yes	Sample homogenization	Yes
Storage temperature	-80 °C	Sample homogenization solvent	First: Phosphate Buffer Saline (PBS); Second: Isopropanol

Lipid Class Descriptions

1) FA[M-H]- / Lipid identification

Lipid class	FA	Limit of detection	Signal theshold
MS Level for identification	MS1	RT verified by standard	Yes
Identification level	sn Position	Separation of isobaric/isomeric interferece confirmed	Yes
Polarity mode	Negative	Model for separation prediction	Yes
Type of negative (precursor)ion	[M-H]-	Additional dimension/techniques	-
Isotope correction at MS1	Type 2	Lipid Identification Software	Homemade
MS1 verified by standard	Yes	Data manipulation	-
Background check at MS1	Yes	Nomenclature for intact lipid molecule	Yes
Did you presume assumptions for identification?	No	Further identification remarks	Relative comparison of FA levels obtained through a full scan untargeted LC-HRMS profiling and in-house library matching based on accurate mass and retention time (AMRT)
Check on:	In-source fragmentation		

1) FA[M-H]- / Lipid quantification

Quantitative	No	Batch correction	No
Normalization to reference	Yes	Further quantification remarks	Targeted data mining and FA annotation in patient samples performed by matching the accurate mass and retention time (AMRT) of 25 pure authentic FA standards (Sigma-Aldrich) analyzed under the same analytical conditions

Contents of Report

Created by https://lipidomicstandards.org, version v2.4.0

Separation Workflow 1
Overall study design . . . 1
Lipid extraction . . . 1
Analytical platform . . . 1
Quality control . . . 2
Method qualification and validation . . . 2
Reporting . . . 2

Sample Descriptions 2
TS-FFA-072024_number / Human / Tissues (e.g., liver, heart, brain) . . . 2

Lipid Class Descriptions 3
1) FA[M-H]- / Lipid identification . . . 3
1) FA[M-H]- / Lipid quantification . . . 3

Separation Workflow

Overall study design

Title of the study	Depletion-dependent Activity-Based Protein Profiling coupled to SWATH/DIA Mass Spectrometry detects serine hydrolase lipid remodeling in lung adenocarcinoma progression		
Document creation date	02/10/2025	Corresponding Email	tatjana.sajic@chuv.ch
Principal investigator	Tatjana Sajic	Is the workflow targeted or untargeted?	Targeted
Institution	CHUV	Clinical	Yes

Lipid extraction

Extraction method	1-phase system	Were internal standards added prior extraction?	Yes
pH adjustment	Phosphate Buffer Saline	Special conditions	intensive mixing
1-phase system	Isopropanol	Derivatization	No

Analytical platform

Ionizaton additives	Ammonium acetate	Ion source	ESI
Number of separation dimensions	One dimension	MS Level	MS1
Separation type 1	ultra-high performance liquid chromatography (UHPLC)	Mass resolution for detected ion at MS1	High resolution
Separation mode 1 (generic)	Zorbax Eclipse Plus C18 (1.8 m, 100 mm × 2.1 mm I.D. column)	Resolution at m/z 200 at MS1	60000
Detector	Mass spectrometer	Mass accuracy in ppm at MS1	5
MS type	Orbitrap	Recording mode of raw data at MS1	Centroid mode
MS vendor	Thermo	Was/Were additional dimension/techniques used	No

Quality control

Blanks	Yes	Quality control	Yes
Type of Blanks	Extraction blank, Solvent blank, Internal standard blank	Type of QC sample	Commercial sample, Sample pool, Reference material

Method qualification and validation

Method validation	Yes	Precision	Yes
Lipid recovery	Yes	Accuracy	Yes
Dynamic quantification range	Yes	Guidelines followed	EMA
Limit of quantitation (LOQ)/Limit of detection (LOD)	Yes		

Reporting

Are reported raw data uploaded into repository?	Yes	Summary data	Quantification and identification data
Link to repository / ID to entry	10.5281/zenodo.14841692	Raw data upload	Yes
Are metadata available?	Available on request	Additional comments	Absolute quantification by using 4 stable isotope labelled internal standards and Calibration Curve Preparation.

Sample Descriptions

TS-FFA-072024_number / Human / Tissues (e.g., liver, heart, brain)

Perfusion	No	Storage time (month)	120
Storage and collection conditions	Available	Freeze-thaw cycles	0
Provided preanalytical information	Time to freeze (min), Storage time (month), Freeze-thaw cycles	Additives	None
Temperature handling original sample	-20 °C	Were samples stored under inert gas?	No
Instant sample preparation	Yes	Additional preservation methods	No
Time to freeze (min)	5	Biobank samples	Yes
Snap freezing in liquid N2	Yes	Sample homogenization	Yes
Storage temperature	-80 °C	Sample homogenization solvent	1.Phosphate Buffer Saline 2. Isopropanol

Lipid Class Descriptions

1) FA[M-H]- / Lipid identification

Lipid class	FA	Check on:	-
MS Level for identification	MS1	Limit of detection	Signal theshold
Identification level	Double bond position	RT verified by standard	Yes
Polarity mode	Negative	Separation of isobaric/isomeric interferece confirmed	Yes
Type of negative (precursor)ion	[M-H]-	Model for separation prediction	Yes
Isotope correction at MS1	Type 2	Additional dimension/techniques	-
MS1 verified by standard	Yes	Lipid Identification Software	Xcalibur™ Software
Background check at MS1	Yes	Data manipulation	Centroiding, Lock mass correction, Background subtraction
Did you presume assumptions for identification?	Yes	Nomenclature for intact lipid molecule	No
Which assumptions were presumed?	We chose palmitic acid, on the basis of our hypotheses in the proteomics experiments, and three FA (palmitoleic, oleic, and eicosapentaenoic) that exhibited statistically significant changes in the relative comparisons of FA profiles in the discovery experiment	Further identification remarks	Internal standard and calibration curve preparation for absolute quantification

1) FA[M-H]- / Lipid quantification

Quantitative	Yes	Type I isotope correction	Yes
MS Level for quantification	MS1	Limit of quantification	Signal theshold
Internal lipid standard(s) MS1		Normalization to reference	Yes

Internal standard	Endogenous subclass
Palmitoleicacid-d13	LarodanAG(Solna,Sweden)
Oleicacid-d9	LarodanAG(Solna,Sweden)
Eicosapentaenoicacid-d5	LarodanAG(Solna,Sweden)
Hexadecenoicacid-d3	LarodanAG(Solna,Sweden)

Type of quantification	Calibration line	Lipid Quantification Software	Xcalibur™ Software
Type of calibration line	Solvent based	Batch correction	No
Species calibration line	Sigma-Aldrich: Oleic acid (C18:1 (n-9)), Palmitic acid(C16:0), Palmitoleic acid(C16:1 (n-7)), Eicosapentaenoic (20:5 (n-3)) acid	Further quantification remarks	Absolute quantification by the calibration curves (prepared with authentic non-labeled pure standards, Sigma-Aldrich) and isotopically labelled or deuterated internal standards (IS)
Response correction	Response model		
