# Supplementary material for: Depletion-dependent activity-based protein profiling using SWATH/DIA-MS detects serine hydrolase lipid remodeling in lung adenocarcinoma progression
Source: Nat Commun. 2025 May 27;16:4889. doi: 10.1038/s41467-025-59564-x (PMC12117057; doi:10.1038/s41467-025-59564-x)
Supplement: Supplementary file 2 — Reporting Summary [file 41467_2025_59564_MOESM2_ESM.pdf]

nature portfolio

Corresponding author(s): Tatjana Sajic, Sven Hillinger and Ruedi Aebersold

Last updated by author(s): April 8, 2025.

Reporting Summary

Nature Portfolio wishes to improve the reproducibility of the work that we publish. This form provides structure for consistency and transparency in reporting. For further information on Nature Portfolio policies, see our Editorial Policies and the Editorial Policy Checklist.

Statistics

For all statistical analyses, confirm that the following items are present in the figure legend, table legend, main text, or Methods section.

n/a	Confirmed	
☐	☒	The exact sample size (*n*) for each experimental group/condition, given as a discrete number and unit of measurement
☐	☒	A statement on whether measurements were taken from distinct samples or whether the same sample was measured repeatedly
☐	☒	The statistical test(s) used AND whether they are one- or two-sided<br>*Only common tests should be described solely by name; describe more complex techniques in the Methods section.*
☐	☒	A description of all covariates tested
☐	☒	A description of any assumptions or corrections, such as tests of normality and adjustment for multiple comparisons
☐	☒	A full description of the statistical parameters including central tendency (e.g. means) or other basic estimates (e.g. regression coefficient) AND variation (e.g. standard deviation) or associated estimates of uncertainty (e.g. confidence intervals)
☐	☒	For null hypothesis testing, the test statistic (e.g. *F*, *t*, *r*) with confidence intervals, effect sizes, degrees of freedom and *P* value noted<br>*Give P values as exact values whenever suitable.*
☒	☐	For Bayesian analysis, information on the choice of priors and Markov chain Monte Carlo settings
☒	☐	For hierarchical and complex designs, identification of the appropriate level for tests and full reporting of outcomes
☐	☒	Estimates of effect sizes (e.g. Cohen's *d*, Pearson's *r*), indicating how they were calculated

Our web collection on statistics for biologists contains articles on many of the points above.

Software and code

Policy information about availability of computer code

Data collection	This study focused on analysis of lung human samples of patients with lung carcinoma. Raw data of all MS measurements of respective bio-samples are deposited and available on the PRIDE archive Project accession: PXD019357 (https://www.proteomexchange.org/). Targeted lipidomics data are available via Zenodo https://doi.org/10.5281/zenodo.14841692. Lipidomics Minimal Reporting Checklist (https://lipidomicstandards.org/) for the two types of FA measurements are available in the Supplementary Material. Cancer genomics analysis is based on data generated by the TCGA Research Network. Cancer genomics data sets are publicly available from the TCGA Research Network http://cancergenome.nih.gov/. TAMRA fluorescence output files are available in the 10.6084/m9.figshare.28755566. Source data are provided with this paper.
Data analysis	Proteomics Data analysis performed via OpenSWATH integrated in the Euler portal workflow (06/08/2018) and integrated in OpenMS (https:openms.de/) (Rost, H.L., et al.2014).OpenSWATH workflow integrates the feature alignment algorithm TRIC (Rost, H.L., et al.,2016). Raw SWATH sample files were converted into the mzXML format ProteoWizard (version 3.0.331655). TraML DIA Library format generated using the OpenMS tool ConvertTSVToTraML (version 1.10.0) and the decoy transition groups were generated based on shuffled sequences by the OpenMS tool OpenSwathDecoyGenerator (version 1.10.0)<br><br>Lipidomics Raw LC-HRMS data (experiment1) were verified for quality using MassHunter Agilent Technologies software (version10.0) and processed using Agilent Profinder 8.0 software. Targeted lipidomics data for absolute quantification reported by Thermo TraceFinder software, optimized for clinical research.<br><br>Statistical analysis performed with R version 3.6.1 .The R software tool is available from the CRAN repository: https://cran.r-project.org/.

(R packages used: MSstats.daily v. 2.3.5, SWATH2stats (version 1.8.1) , plyr version 1.8.6, ggplot2 version 3.3.5, mixOmics version 6.10.9, 'pheatmap' version 1.0.12, DOSE(Yu et al. 2015) package). For the reported statistical analyses of paired extracts, we used also in-house written R code available via 10.6084/m9.figshare.28755566.

Web-tools:
Cytoscape3.6.0 (https://www.cytoscape.org/)
Web tool for phylogenetic tree visualization: https://itol.embl.de/
Panther.db. https://www.pantherdb.org/
Merops database (https://www.ebi.ac.uk/merops/)
Protein-protein interactions (PPIs) in human assessed from an Integrated Interactions Database (http://ophid.utoronto.ca/iid.)

For manuscripts utilizing custom algorithms or software that are central to the research but not yet described in published literature, software must be made available to editors and reviewers. We strongly encourage code deposition in a community repository (e.g. GitHub). See the Nature Portfolio guidelines for submitting code & software for further information.

Data

Policy information about availability of data

All manuscripts must include a data availability statement. This statement should provide the following information, where applicable:

- Accession codes, unique identifiers, or web links for publicly available datasets
- A description of any restrictions on data availability
- For clinical datasets or third party data, please ensure that the statement adheres to our policy

Proteomic Datasets analyzed here are available via PRIDE archive Project accession: PXD019357. (Reviewer account: Username: reviewer82442@ebi.ac.uk, Password: BVcHwScd) .Lipidomic raw data complementing the primary proteomic data and related to relative and absolute FA measurements are available via Zenodo https://doi.org/10.5281/zenodo.14841692. Lipidomics Minimal Reporting Checklist (https://lipidomicstandards.org/) for the two types of FA measurements are available in the Supplementary Data. Cancer genomics data sets are publicly available from the TCGA Research Network http://cancergenome.nih.gov/. Proteases and protease inhibitors annotation from Merops (https://www.ebi.ac.uk/merops/) and Panther.db. https://www.pantherdb.org/. Protein-protein interactions (PPIs) in human assessed from an Integrated Interactions Database (http:ophid.utoronto.caiid.). List of e harmful metals identified in tobacco smoke available by The Food and Drug Administration (FDA)(www.fda.gov/tobacco-products/) . Biochemical validation data are available in the main text or the supplementary material. Raw gel pictures can be found in the Supplementary Materials. The individual morphological tissue data for each patient are available upon request.

Research involving human participants, their data, or biological material

Policy information about studies with human participants or human data. See also policy information about sex, gender (identity/presentation), and sexual orientation and race, ethnicity and racism.

Reporting on sex and gender	In this study we include both, male and female individuals. The gender status is treated as categorical variables that have two possible outcomes, 0 or 1 (e.g., male vs female) and the analysis accounted for patient confounders such as age, sex, smoking status.
Reporting on race, ethnicity, or other socially relevant groupings	N/A
Population characteristics	The analysis accounted for patient confounders such as age, sex, smoking status. The gender and smoking status are treated as categorical variables that have two possible outcomes, 0 or 1 (e.g., male/female or smoking/non-smoking). Patients'age in years was a continuous variable. We also reported disaggregated data and non-adjusted analysis in the Supplementary Material (Supplementary Table 1, Source Data Table 1).
Recruitment	Clinical cohort of patients who had undergone surgical lung resection at the University Hospital Zurich (UHZ) between 2005 and 2013.
Ethics oversight	Cantonal Ethics Committee Zurich (KEKZH) has approved all procedures<br>involving human material and each patient has signed an informed consent form<br>(KEK-ZH-No. 2020-02566).

Note that full information on the approval of the study protocol must also be provided in the manuscript.

Field-specific reporting

Please select the one below that is the best fit for your research. If you are not sure, read the appropriate sections before making your selection.

[x] Life sciences [ ] Behavioural & social sciences [ ] Ecological, evolutionary & environmental sciences

For a reference copy of the document with all sections, see nature.com/documents/nr-reporting-summary-flat.pdf

Life sciences study design

All studies must disclose on these points even when the disclosure is negative.

Sample size	Sample size corresponded to 32 distinct lung tissues collected as either tumor or surrounding, non-neoplastic lung parenchyma. For discovery cohort we analyzed 24 distinct samples. For validation cohort we collected novel tissue cuts from 28 distinct samples, of which 8 samples were independent from discovery cohort. For each experiment we used 5-9 samples / group- tissue types. Genetic polymorphisms of SHs enzymes was explored within The Cancer Genome Atlas (TCGA) datasets and involved 230 LUAD cancers.
Data exclusions	We did not exclude the data. We used the measurements of all available samples and patients. Each sample unit (biological sample) was a pool of an average of 30 (12-75) consecutive tissue microsections to ensure a representative sample. We found high level of data reproducibility between different cuts within the same individual tissue bio-sample.
Replication	We verified "whole method" reproducibility via parallel measurements of several distinct tissue cuts from the same bio specimen. To monitor MS-data reproducibility , we used replicated MS injections from the same extract. The technical replicates were injected at different time points across the respective MS injection queue to cover all MS variations. We also include “well defined quality controls” after each of three MS injections of tested patient samples in order to monitor instrument signal variability and MS sensitivity in total experiment. For commercial activity assays of selected proteases, measurements were performed in triplicates that are averaged.
Randomization	The samples were injected randomly across the respective MS injection queue . We did not apply sample randomization in the process of sample extraction.
Blinding	The injection of extracted samples into the LC-MS was a completely blind experiment and the MS operators did not have access to data labels or group assignments. Researchers and clinicians were not blinded during certain steps of data collection.

Reporting for specific materials, systems and methods

We require information from authors about some types of materials, experimental systems and methods used in many studies. Here, indicate whether each material, system or method listed is relevant to your study. If you are not sure if a list item applies to your research, read the appropriate section before selecting a response.

Materials & experimental systems

n/a	Involved in the study	
☐	☒	Antibodies
☒	☐	Eukaryotic cell lines
☒	☐	Palaeontology and archaeology
☒	☐	Animals and other organisms
☐	☒	Clinical data
☒	☐	Dual use research of concern
☒	☐	Plants

Methods

n/a	Involved in the study	
☒	☐	ChIP-seq
☒	☐	Flow cytometry
☒	☐	MRI-based neuroimaging

Antibodies

Antibodies used	Anti-SCD1 antibody [CD.E10] (ab19862) from Abcam; Loading controls: GAPDH (MAB374, Sigma Aldrich); b-Actin (AC-15, Sigma Aldrich)
Validation	Anti-SCD1 antibody : https://www.abcam.com/products/primary-antibodies/scd1-antibody-cde10-ab19862.html. Publication: PMID: 33511729

Clinical data

Policy information about clinical studies

All manuscripts should comply with the ICMJE guidelines for publication of clinical research and a completed CONSORT checklist must be included with all submissions.

Clinical trial registration	Cantonal Ethics Committee Zurich (KEKZH) has approved all procedures involving human material and each patient has signed an informed consent form (KEK-ZH-No. 2020-02566).
Study protocol	Trial protocol available upon request from University Hospital Zurich (UHZ).
Data collection	Patients who had undergone surgical lung resection at the University Hospital Zurich (UHZ) between 2005 and 2013.
Outcomes	Clinical data record of respective patients segregated individuals with survival period superior to 5 years or 1 year, respectively. Initially, clinical pathologists predicted the patient outcomes in accordance with the 6th TNM classification guidelines (tumor size, lymph node status, and metastases).

Plants

Seed stocks	*Report on the source of all seed stocks or other plant material used. If applicable, state the seed stock centre and catalogue number. If plant specimens were collected from the field, describe the collection location, date and sampling procedures.*
Novel plant genotypes	*Describe the methods by which all novel plant genotypes were produced. This includes those generated by transgenic approaches, gene editing, chemical/radiation-based mutagenesis and hybridization. For transgenic lines, describe the transformation method, the number of independent lines analyzed and the generation upon which experiments were performed. For gene-edited lines, describe the editor used, the endogenous sequence targeted for editing, the targeting guide RNA sequence (if applicable) and how the editor was applied.*
Authentication	*Describe any authentication procedures for each seed stock used or novel genotype generated. Describe any experiments used to assess the effect of a mutation and, where applicable, how potential secondary effects (e.g. second site T-DNA insertions, mosiacism, off-target gene editing) were examined.*
