# Supplementary material for: Depletion-dependent activity-based protein profiling using SWATH/DIA-MS detects serine hydrolase lipid remodeling in lung adenocarcinoma progression
Source: Nat Commun. 2025 May 27;16:4889. doi: 10.1038/s41467-025-59564-x (PMC12117057; doi:10.1038/s41467-025-59564-x)
Supplement: Supplementary file 3 — Description of Additional Supplementary Files [file 41467_2025_59564_MOESM3_ESM.pdf]

Description of Additional Supplementary Files

File Name: **Supplementary Data 1**

Description: List of 12,653 peptide transition groups available for monitoring 3593 unique proteotypic SH peptides of 215 SHs, related to Supplementary Fig. 1

(A manually compiled total reference list of 335 SHs in the source data.)

File Name: **Supplementary Data 2**

Description: Reference set of internal standard peptides (ISPs) related to Supplementary Fig. 2

File Name: **Supplementary Data 3**

Patient Information

File Name: **Supplementary Data 4**

Description: List of 131 SHs measured in the LUAD study and their corresponding 793 proteotypic peptides (916 peptide transitions) identified by SWATH-MS at peptide FDR <0.001, related to Figure 3.

File Name: **Supplementary Data 5**

Description: List of 2078 Proteins - 1st degree interactors of 72 SH (RADDi, KS<=0.05), related to Figure 5

File Name: **Supplementary Data 6**

Description: List of 978 Proteins co-depleted with the active SHs. 933 out of 978 proteins are annotated as 1st or 2nd degree interactors in Integrated Interactions Database.

File Name: **Supplementary Data 7**

Description: The binary enzyme:protein interactions across co-depleted proteome based on canonical correlation analysis (CCA) via DIABOLO data integration tool, related to Figure 5.

File Name: **Supplementary Data 8**

Description: Profiling of FA levels in patient tumor lysates, related to Figure 6.

File Name: **Supplementary Data 9**

Description: Original output values of respective metals (Conc. [ng/ml], Conc. RSD and intensity (CPS), related to Figure 7.
